# Photoresponsive Ru Complex–Gold Nanoparticle Hybrids for Theranostics: A Theoretical Study of Electronic Structure and Luminescence-Based Detection

**DOI:** 10.3390/molecules30224432

**Published:** 2025-11-16

**Authors:** Niq Catevas, Athanassios Tsipis

**Affiliations:** Laboratory of Inorganic Chemistry, Department of Chemistry, University of Ioannina, 45110 Ioannina, Greece; n.katevas@uoi.gr

**Keywords:** ruthenium nitrosyl complexes, gold nanoclusters, DFT, TDDFT, NBO, ETS–NOCV, NO photorelease, NIR emission, theranostics

## Abstract

Photoactivatable nitric oxide donors (photoNORMs) are promising agents for controlled NO release and real-time optical tracking in biomedical theranostics. Here, we report a comprehensive density functional theory (DFT) and time-dependent DFT (TDDFT) study on a series of hybrid ruthenium–gold nanocluster systems of the general formula [(L)Ru(NO)(SH)@Au_20_], where L = salen, bpb, porphyrin, or phthalocyanine. Structural and bonding analyses reveal that the Ru–NO bond maintains a formal {RuNO}^6^ configuration with pronounced Ru → π*(NO) backbonding, leading to partial reduction of the NO ligand and an elongated N–O bond. Natural Bond Orbital (NBO), Natural Energy Decomposition Analysis (NEDA), and Extended Transition State–Natural Orbitals for Chemical Valence (ETS–NOCV) analyses confirm that Ru–NO bonding is dominated by charge-transfer and polarization components, while Ru–S and Au–S linkages exhibit a delocalized, donor–acceptor character coupling the molecular chromophore with the metallic cluster. TDDFT results reproduce visible–near-infrared (NIR) absorption features arising from mixed metal-to-ligand and cluster-mediated charge-transfer transitions. The calculated zero–zero transition and reorganization energies predict NIR-II emission (1.8–3.8 μm), a region of high biomedical transparency, making these systems ideal candidates for luminescence-based NO sensing and therapy. This study establishes fundamental design principles for next-generation Ru-based photoNORMs integrated with plasmonic gold nanoclusters, highlighting their potential as multifunctional, optically trackable theranostic platforms.

## 1. Introduction

Nitric oxide (NO), although known to be a very reactive [1] and toxic gas [2], plays a crucial role in many biological processes, including cardiovascular regulation [3], antimicrobial treatments [4], and cancer therapy [5,6,7,8,9]. The controlled, photo-triggered release of NO has motivated the development of photoactivatable NO-releasing molecules, the so-called photoNORMs [10,11,12]. Early examples, such as Sodium Nitroprusside (SNP), suffered from limited photostability, degrading upon irradiation to yield toxic photoproducts [13]. Ruthenium complexes have emerged as more robust photoNORM candidates, offering enhanced stability and better biological tolerance [14,15,16,17]. Despite these advantages, conventional Ru–NO complexes face intrinsic limitations: low stability of the Ru–NO bond under physiological conditions, modest quantum yields, and the requirement for high-energy UV light to induce NO release [13]. To address these limitations, various strategies have been explored, including the use of dye-sensitized ligands to extend absorption into the visible range [18,19,20,21], as well as dinuclear Ru (II) systems with pyrazine bridging ligands [22,23]. These approaches enhance photorelease efficiency, yet challenges remain in fully optimizing quantum yields, spectral overlap, and biocompatibility. An emerging and largely unexplored strategy involves hybridizing Ru complexes with nanoparticles (transition metal complex/nanoparticle (TMC/NP) materials), in which a transition metal complex is anchored to a nanoparticle via a suitable ligand [24]. This design can facilitate electronic interactions between the Metal-to-Ligand Charge Transfer (MLCT) bands of the complex and the localized surface plasmon resonance (LSPR) of the nanoparticle, leading to phenomena such as enhanced two-photon absorption [25], increased singlet oxygen generation [26], and elevated fluorescence quantum yields [27]. Such hybrid systems have shown promise in multi-channel luminescence imaging [28] and photodynamic therapy, where nanoparticles act as carriers for photosensitizing TMCs [29]. Their water solubility, tunable coordination environment, and low toxicity make them attractive candidates for biomedical applications, including as photoNORMs [25,30]. A critical limitation in NO-based therapies remains the lack of real-time monitoring of NO’s release and distribution. Here, the photophysical properties of TMC/NP hybrids present a unique opportunity for luminescence-based imaging, offering insight into molecular location, activation, and functionality during therapy. Atomically defined gold nanoclusters provide a particularly suitable platform for these hybrids [31]. Au_20_ clusters are computationally tractable and chemically meaningful: they serve as prototypical models of extended Au surfaces [32,33] and have been experimentally isolated and functionalized [34,35]. Their stability, well-defined icosahedral geometry, and amenability to thiol-based anchoring allow the design of discrete, hybrid Ru–Au_20_ complexes that are both theoretically insightful and potentially synthesizable. Guided by these considerations, we investigated a series of Ru–NO/Au_20_ hybrid complexes of the general formula [(L)Ru(NO)(SH)@Au_20_], where L = *N*,*N*′-bis(salicylidene)ethylenediamine (Salen), 1,2-bis(pyridine-2-carboxamido)benzenate (bpb), porphyrin (Porph), or phthalocyanine (Pc). These complexes exemplify the emerging class of hybrid transition metal complex/nanoparticle (TMC/NP) materials [24], where molecular photoactive units are anchored directly to gold clusters via thiolate bridges. The goal of this study is to explore how the electronic structure and photophysical behavior of Ru–NO units are modified when the complex is anchored to gold NPs. To keep the systems under investigation computationally tractable, we modeled the bulk gold NPs using a small Au_20_ NP. Note that these systems could also be potentially synthesized (vide supra) as is, yielding hybrid complexes with intriguing properties. We chose four representative equatorial ligand environments (salen, bpb, porphyrin, and phthalocyanine) to examine how ligand electronics influence the Ru–NO bond and the interaction with the gold cluster. Our underlying hypothesis is that the Ru–Au linkage can introduce new charge-transfer pathways and alter the Ru → NO backdonation, with possible consequences for N–O activation, excited-state ordering, and NIR absorption/emission. These aspects are directly relevant to the design of Ru-based photoNORM compounds. By analyzing the computed bonding indicators and excited-state properties, we aimed to identify structural and electronic features that could be beneficial for NO photorelease as well as for luminescent detection of NO. To the best of our knowledge, this is the first computational study in which a transition metal nitrosyl complex is covalently combined with a gold nanocluster and examined as a potential photoNORM system and luminescent NO probe. Such Ru–NP hybrid architectures have not previously been explored in this context.

## 2. Results and Discussion

### 2.1. Structural Properties

**Figure 1:**
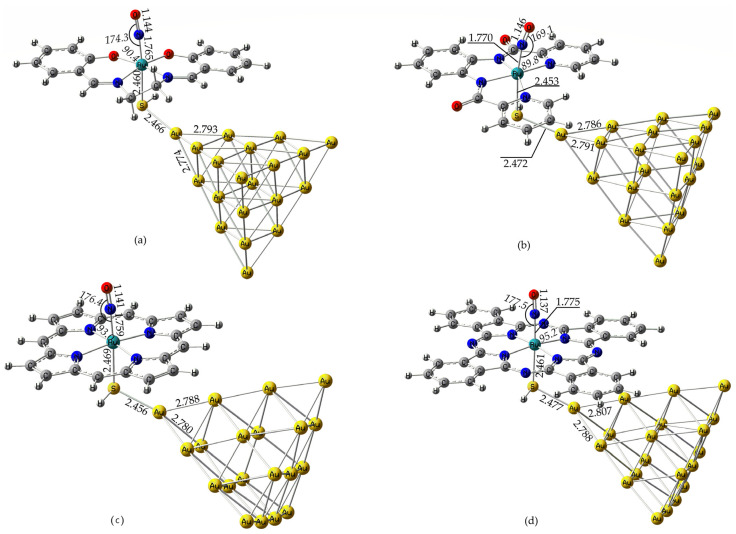
Optimized geometries of [(Salen)Ru(NO)(SH)], (**a**) [(bpb)Ru(NO)(SH)], (**b**) [(Porph)Ru(NO)(SH)], (**c**) and [(Pc)Ru(NO)(SH)], (**d**) along with selected structural parameters at the PBE0/LanL2DZ(Ru)U6-31G(d,p)(E) level in water solvent.

To rationalize the photophysical and sensing behavior of the [(L)Ru(NO)(SH)@Au_20_] hybrid complexes, we first examined their structural, bonding and electronic properties. In Figure 1 are depicted the optimized geometries of the Ru (II) under study, along with selected structural parameters. Based on the oxidation state of Ru (II) and the formal NO^+^ assignment, the complexes are best described as {RuNO}^6^ species according to the Enemark–Feltham notation.

As shown in Figure 1, the nearly linear Ru–N–O bond angles (169–178°) observed in these Ru (II) complexes support a formal NO^+^ assignment, consistent with a {RuNO}^6^ configuration as defined by the Enemark–Feltham notation. This is further corroborated by the relatively short N-O bond lengths, falling in the range 1.137–1.146 Å, usually observed for cationic NO^+^ coordinated to low-spin d^6^ Ru (II) complexes [36,37]. Figure S1 in the Supplementary Materials presents the simulated IR spectra of the Ru (II) nitrosyl complexes studied. The most intense absorption band, observed in the 1970–2020 cm^−1^ region, is attributed to the symmetric stretching vibration of the N–O bond *V*_s_(N–O) of the nitrosyl ligand. This feature is characteristic of a linearly coordinated NO^+^ moiety, indicative of strong N–O multiple bonding. We note that metal–nitrosyl *V*_s_(N–O) stretching frequencies, unlike organic nitroso N–O stretches, typically appear in the 1800–2100 cm^−1^ region due to the linear Ru–NO^+^ bonding motif and strong π backbonding [36]. Our calculated values (1970–2020 cm^−1^) are in excellent agreement with these established benchmarks. To further validate this, we performed optimization and vibrational analysis of two benchmark Ru (II) octahedral complexes, i.e., the [(NH_3_)_5_Ru(NO)]^3+^ and the [(CN)_5_Ru(NO)]^2−^, employing our computational protocol (PBE0/LanL2DZ(Ru)U6-31G(d,p)(E)/PCM(Water)). Again, the ν_s_(N–O) stretching frequencies were found at 2089 and 1905 cm^−1^ for the [(NH_3_)_5_Ru(NO)]^3+^ and the [(CN)_5_Ru(NO)]^2−^ complexes, respectively, in very good agreement with previously reported results [36]. Due to the large size of the Ru–NO–Au_20_ hybrid complexes and the well-localized character of the ν_s_(N–O) mode, a full Potential Energy Distribution (PED) analysis was not performed. The assignment of the nitrosyl stretching vibration is unambiguous and consistent with benchmark Ru–NO systems and established experimental ranges (ca. 1850–2100 cm^−1^).

Note that there is an excellent correlation between the *R*_e_(N-O) bond length and the ν_s_(N–O) for the four Ru (II) complexes under study, as expected. Thus, the *R*_e_(N-O) bond lengths follow the order [(Salen)Ru(NO)(SH)] < [(bpb)Ru(NO)(SH)] < [(Porph)Ru(NO)(SH)] < [(Pc)Ru(NO)(SH)], while the ν_s_(N–O) stretching frequencies follow the opposite order. Nevertheless, the effect of the equatorial ligand on the *R*_e_(N-O) and ν_s_(N–O) is relatively minor. The same is also observed for the ν_s_(N–O) frequency intensity, where, again, the effect of the equatorial ligand is only minor. Nevertheless, in all cases, the N-O bond length in the Ru (II) complexes under study was found to be elongated as compared to that found for the free NO^+^ cation at the same level of theory (1.07 Å). Also, the ν_s_(N–O) frequency calculated for the free NO^+^ cation was found to be much higher (2555 cm^−1^) than those found for the Ru (II)–Au_20_ hybrid complexes, consistent with the elongated N-O bond length of the axial NO ligand observed for the latter.

### 2.2. Electronic and Bonding Properties

The electronic and bonding properties of the [(L)Ru(NO)(SH)@Au_20_] Ru (II) hybrid octahedral complexes were further scrutinized upon employing a multitude of methods, such as NBO analysis, NEDA, and ETS-NOCV methods.

#### 2.2.1. NBO Analysis

**Figure 2:**
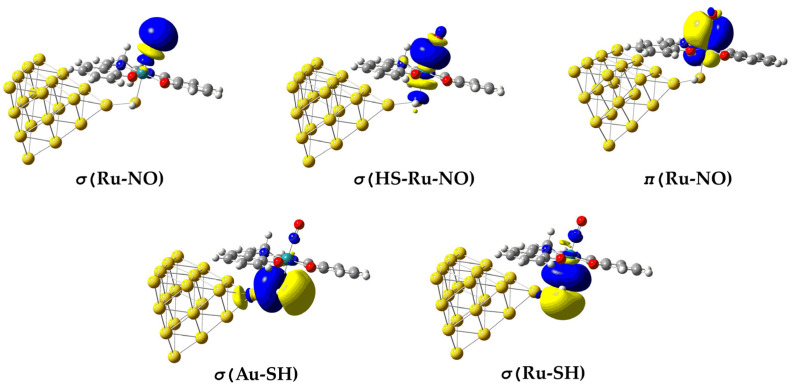
The 3D surfaces of BD NBOs relevant to bonding in the Au_20_-(H)S-Ru-NO framework in the [(Salen)Ru(NO)(SH)@Au_20_] complex.

In Figure 2 are depicted the 3D surfaces of the NBOs, relevant to the bonding properties in the representative [(Salen)Ru(NO)(SH)] complex. Similar NBOs are also observed for the rest of the Ru hybrid complexes under study. Also, in Table 1 are given selected NBO-derived parameters describing the electronic properties of the Ru hybrids under study. In terms of NBO analysis, the Ru–NO bond in the [(Salen)Ru(NO)(SH)] complex is described by two *σ*-bonding (BD) NBOs—namely, the *σ*(Ru–NO) and the *σ*(HS-Ru–NO)—and one π BD NBO, π(Ru–NO). The *σ*(Ru–NO) orbital arises from a linear combination of an spd-type hybrid orbital (*h*_Ru_) on Ru and an sp-type hybrid orbital (*h*_N_) on the N atom of the axial nitrosyl ligand. However, the Ru-NO bond exhibits a relatively weak covalent component, as reflected by the small WBI value being less than 0.5 (Table 1). Obviously, the nature of the Ru-NO bond is complex, arising also from electrostatic or even donor–acceptor dative interactions. The existence of the former is clearly verified by the opposite natural charges calculated for the Ru metal center (*Q*_Ru_) and the N donor atom (*Q*_N_) of the NO ligand being 0.695 ∣e∣ and −0.464 ∣e∣, respectively (Table 1). The dative nature of the Ru-NO bond is also verified based upon the NEDA and ETS-NOCV analysis methods (vide infra). It should also be noted that the *σ*(HS-Ru–NO) BD NBO is delocalized over the entire HS-Ru-NO framework, probably due to the presence of the Au_20_ NP. On the other hand, the *π*(Ru–NO) DB NBO (Figure 2) is a linear combination of d-type *h*_Ru_ with *p*-type *h*_N_.

Remarkably, no explicit two-center Ru–S- or Au–S-bonding NBOs were detected, consistent with a delocalized nature of these linkages. In the *σ*(Ru–SH) and *σ*(Au–SH) BD NBOs (Figure 2), the sulfur lone pairs act as donors toward low-lying, metal-centered acceptor orbitals on Ru and the Au_20_ cluster, producing strong LP(S) → Ry*(Ru/Au) delocalization interactions (LP = lone pair orbital, Ry = Rydberg orbital) with stabilization energies of several tens of kilocalories per mole. This pattern indicates that the Ru–S and Au–S connections are dative and multicenter, rather than classical shared-electron bonds. The electron density associated with the Au_20_ cluster is further delocalized over multiple Au atoms, forming a quasi-metallic manifold that electronically couples the Ru–NO chromophore to the gold surface. It should be noted, however, that the Ru–S bond is expected to have a significant electrostatic component, analogous to that observed for the Ru-NO bond, since the natural charge (*Q*_S_) on the S donor atom amounts to −0.474. In contrast, the electrostatic component of the S-Au bond, connecting the Au_20_ NP to the Ru metal center, should be marginal due to the very small positive natural charge on the Au atom (*Q*_Au_) directly connected to the S atom amounting to only 0.003 (Table 1).

Finally, inspection of Table 1 reveals that the N–O bond in the coordinated axial nitrosyl ligand is significantly weakened relative to free NO^+^: the WBI(N-O) decreases from 2.792 (free NO^+^, same level of theory) to <2 in the complexes, consistent with substantial Ru → π*(NO) backbonding. This is accompanied by increased π*(NO) occupancy and LP(Ru) → π*(NO) donor–acceptor stabilization, in accordance with the elongation of the N–O distance and the red-shift of the computed *ν*_s_(N-O) observed for these Ru hybrid complexes (vide supra), all indicative of partial reduction (non-innocent behavior) of the NO ligand.

In general, the nature of the equatorial ligand L does not significantly affect the electronic and bonding properties, as could be deduced from the results of the NBO analysis tabulated in Table 1, as well as from the BD NBOs observed for the rest of the [(L)Ru(NO)(SH)@Au_20_] complexes with L = bpb, porph, or pc, being similar to that found for the [(salen)Ru(NO)(SH)@Au_20_] complex and depicted in Figure 2.

#### 2.2.2. NEDA

To further probe the bonding in the Ru (II) [(L)Ru(NO)(SH)@Au_20_] hybrid complexes, we performed Natural Energy Decomposition Analysis (NEDA) on the Ru–NO, Ru–SH, and SH–Au interactions. Table 2 summarizes the NEDA energy components—electrostatics (ES), polarization (POL), charge transfer (CT), and exchange-correlation (XC)—along with the total interaction energy (ΔE) for [(L)Ru(NO)(SH)@Au_20_] hybrid complexes derived from NBO analysis at the PBE0/LanL2DZ(Ru)U6-31G(d,p)(E) level in water solvent. In all cases, the Ru–NO bond is dominated by charge transfer (CT), followed by polarization (POL) and electrostatics (ES), with exchange-correlation (XC) providing a modest contribution. The strength of this interaction is reflected in the highly negative Δ*E* values (ca. −200 to −224 kcal mol^−1^). The Au–SH interactions are considerably weaker (−28 and −18 kcal mol^−1^, respectively), athough they remain CT-dominated. Unlike the Ru–NO bond, the polarization’s contribution to Au–SH is minimal, suggesting a more localized donor–acceptor interaction at the nanoparticle–ligand interface.

A closer inspection of the NEDA results summarized in Table 2 provides deeper insight into the subtle electronic variations induced by the equatorial ligand and the Au_20_ support. Across all [(L)Ru(NO)(SH)@Au_20_] hybrids, the Ru–NO bond is unequivocally dominated by charge-transfer (CT) contributions (≈ −500 kcal mol^−1^), accompanied by substantial polarization (POL) energies (≈ −200 kcal mol^−1^) and modest electrostatic (ES) terms. This CT ≫ POL > ES trend confirms the highly covalent, donor–acceptor character of the Ru–NO linkage, consistent with significant Ru → π*(NO) backbonding revealed by NBO and ETS–NOCV analyses. Among the four ligands, the bpb system exhibits the largest total interaction energy (Δ*E* ≈ −202 kcal mol^−1^), suggesting slightly stronger Ru–NO coupling due to enhanced π-delocalization and ligand field stabilization. In contrast, the phthalocyanine analogue displays a smaller Δ*E* (≈ −181 kcal mol^−1^), reflecting partial electron delocalization over the extended π-system that slightly attenuates direct Ru–NO donation.

For the Ru–S bonds, CT remains the principal stabilizing factor (−160 to −185 kcal mol^−1^), albeit with a comparatively larger polarization and electrostatic component relative to Ru–NO, implying a mixed covalent–ionic character. The magnitude of Δ*E* decreases in the order bpb > salen > pc > porph, in line with the increasing softness of the equatorial donor set. The Au–S interactions are considerably weaker (Δ*E* ≈ −22 to −28 kcal mol^−1^) yet are still governed by CT, underscoring the donor–acceptor nature of the thiolate–gold anchoring. Notably, the phthalocyanine complex shows the most attenuated Au–S interaction, consistent with greater charge delocalization across the macrocyclic π-manifold that diminishes the localized donor capacity at sulfur. Overall, the NEDA analysis highlights a coherent bonding hierarchy—Ru–NO ≫ Ru–S ≫ Au–S—driven predominantly by charge-transfer and polarization effects. These findings substantiate the picture of an electronically coupled Ru–S–Au framework that mediates efficient communication between the photoactive Ru–NO center and the plasmonic nanocluster, a prerequisite for the observed charge-transfer transitions and NIR emission behavior discussed below.

#### 2.2.3. ETS-NOCV

**Figure 3:**
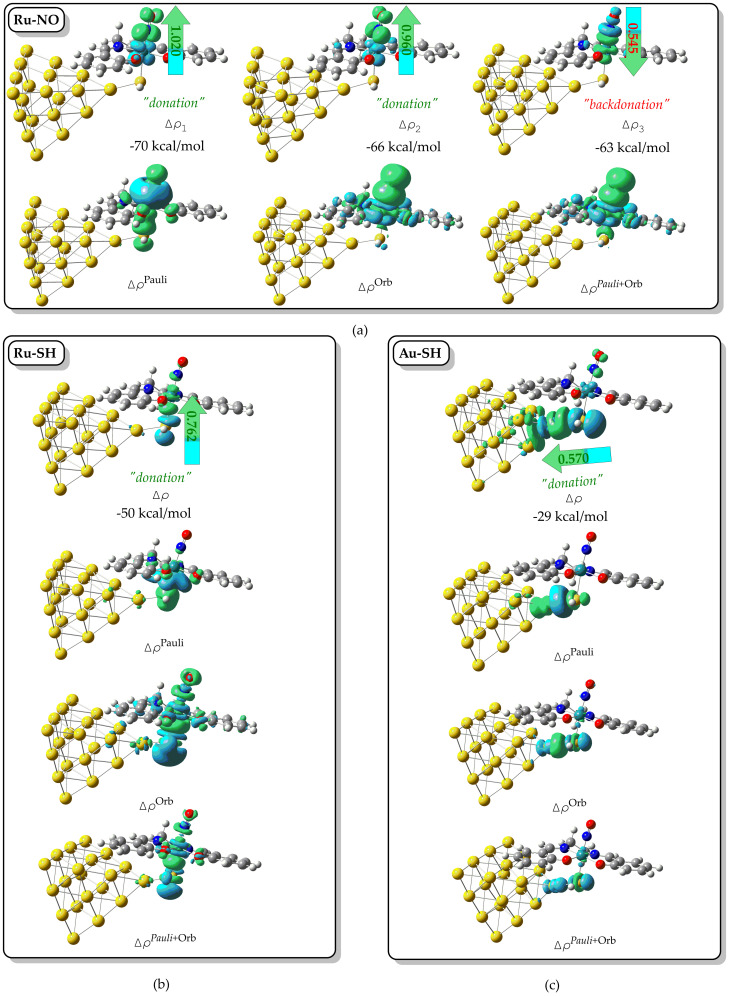
The 3D isosurfaces of selected NOCV pairs and Δ*ρ*^Pauli^, Δ*ρ*^Orb^, and Δ*ρ* deformation density terms calculated for the (**a**) Ru-NO, (**b**) Ru-SH, and (**c**) Au-SH interactions in [(Salen)Ru(NO)(SH)@Au_20_] hybrid complexes (blue and green isosurfaces display decreased and increased electron density regions, respectively).

A powerful approach for dissecting metal–ligand bonding is the ETS–NOCV (Extended Transition State—Natural Orbitals for Chemical Valence) method. We employed this technique to investigate the nature of the Ru–NO, Ru–SH, and Au–SH interactions within the representative [(Salen)Ru(NO)(SH)@Au_20_] hybrid complex. Figure 3 displays the relevant NOCV deformation densities for these bonds. The Ru–NO interaction is characterized by three dominant NOCV pairs with associated orbital interaction energies of −70, −66, and −63 kcal mol^−1^, respectively. These correspond to three principal channels of electron density deformation (Δρ_1–3_, Figure 3a), arising from donor–acceptor interactions between the NO ligand and the Ru center. The first two channels (Δρ_1_ and Δρ_2_) involve σ- and π-donation from the NO lone pair into vacant Ru d-orbitals, with integrated charge transfers of 1.020 and 0.960 electrons, respectively. The third channel (Δρ_3_) represents π-backdonation from filled Ru dπ orbitals into the π* orbital of the NO ligand, with a charge flow of 0.545 |e|.

This combination of strong donation and backdonation supports a synergic bonding picture and aligns with the observed linear Ru–N–O geometry and slightly elongated N–O bond, consistent with a [Ru(II) ← NO^+^] description. Collectively, these three NOCV channels account for ~86% of the total orbital interaction energy (−232 kcal mol^−1^), underscoring their dominant role in bond formation. Notably, the total deformation density (Δρ^Pauli+Orb^ = Δρ^Pauli^ + Δρ^Orb^) closely resembles the orbital-only term (Δρ^Orb^), indicating minimal Pauli repulsion and confirming a strongly donor–acceptor character (Figure 3a). We next examined the Ru–SH and Au–SH interactions, which play a key role in anchoring the complex to the nanoparticle surface and mediating electronic communication relevant for plasmon-enhanced Raman scattering and NO-based holographic response (vide infra). Each interaction is dominated by a single NOCV channel, with orbital stabilization energies of −50 and −29 kcal mol^−1^ for Ru–SH and Au–SH, respectively. As shown in Figure 3b,c, these deformation densities correspond to σ-donation from the sulfur lone pair into vacant d-orbitals on the Ru center and the surface-bound Au atom of the Au_20_ cluster. The respective charge flows of 0.762 and 0.570 electrons reflect a strong donor character at both metal–sulfur interfaces. No π-backdonation components were detected, indicating that both interactions are purely σ-type in nature. Comparable bonding features were observed in the remaining [(L)Ru(NO)(SH)] complexes with L = bpb, porph, or pc, with similar NOCV pair types and deformation patterns. Only minor variations were found in charge flows and interaction energies, suggesting that the overall bonding framework remains largely conserved across all systems studied.

### 2.3. Luminescence-Based Detection

Luminescence of the proposed Ru–NP theranostic hybrids is a central feature offering the ability to monitor them in real time. Accordingly, it is of importance to study their photophysical properties pertaining to either absorption or emission in depth.

#### 2.3.1. Absorption

**Figure 4:**
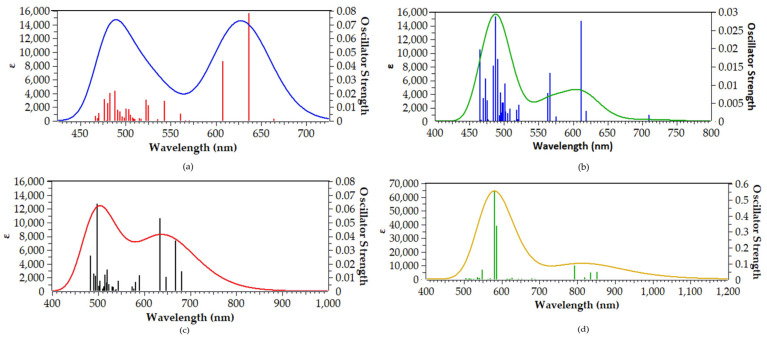
Simulated absorption spectra of (**a**) [(Salen)Ru(NO)(SH)], (**b**) [(bpb)Ru(NO)(SH)], (**c**) [(Porph)Ru(NO)(SH)], and (**d**) [(Pc)Ru(NO)(SH)] calculated at the PBE0/LANL2DZ(Ru)U6-31G(d,p)(E) level in water solvent (fitted with a Gaussian function broadening with full width at half-maximum (FWHM) equal to 0.2 eV or ≈30–50 nm).

To evaluate their optical response under physiologically relevant conditions, we performed TDDFT calculations of 30 singlet–singlet vertical excitations for the [(Salen)Ru(NO)(SH)] complex and its Au_20_-bound hybrid analogues in water solvent. The resulting simulated absorption spectra are depicted in Figure 4.

Before we proceeded in analyzing the spectra obtained for the [(L)Ru(NO)(SH)@Au_20_] hybrid complexes, we set out to benchmark our computational protocol in predicting the UV-Vis spectra of Ru complexes. Since there are no available experimental data for the hybrid complexes under study, we used two other, simpler Ru (II) octahedral complexes, namely, [(CN)_5_Ru(NO)]^2−^ and [(NH_3_)_5_Ru(NO)]^3+^. The simulated absorption spectrum obtained with our computational protocol shows a band at 434 nm for [(CN)_5_Ru(NO)]^2−^, in excellent agreement with the experimentally derived value of 440 nm in aqueous solution reported by Vugman et al. [38] and the value of 444 nm reported by Guenzburger et al. [39]. Also, for the [(NH_3_)_5_Ru(NO)]^3+^ complex, the experimental UV-Vis spectrum shows bands below 300 nm, in line with the simulated spectrum obtained for this complex using our method (the simulated absorption spectra of the Ru (II) benchmark complexes are depicted in Figure S5).

**Figure 5:**
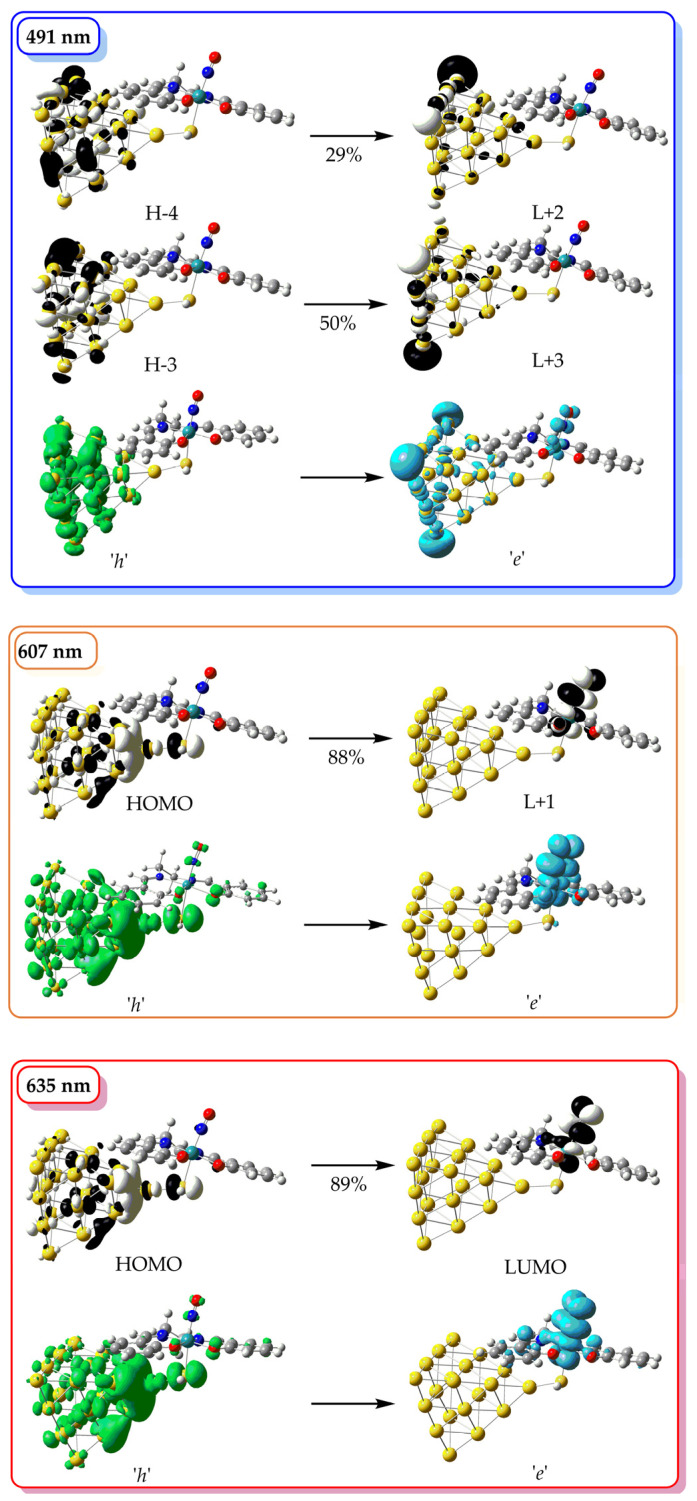
The 3D surfaces of MOs relevant to the most important electronic transitions in the simulated absorption spectra of [(Salen)Ru(NO)(SH)@Au_20_], along with the respective hole (*h*, green surface) and electron (*e*, light blue surface) distributions.

In the simulated absorption spectrum of [(Salen)Ru(NO)(SH)@Au_20_], (blue line in Figure 4, we can observe two bands peaking around at 485 and 625 nm. The high-energy band is comprised of multiple transitions, with the most intense at 491 nm, originating from H–4 → L + 2 and H − 3 → L + 3 excitations (H = HOMO, L = LUMO). All participating molecular orbitals (MOs) are localized on the Au_20_ nanoparticle (Figure 5), and the transition is therefore assigned as metal-centered (MC). The low-energy band is dominated by transitions at 607 and 635 nm, corresponding to H → L + 1 and H → L excitations, respectively. Here, the HOMO is delocalized over the Au_20_ NP and the thiol S atom, while the LUMO and L + 1 are localized on the Ru center and NO ligand, indicating a mixed metal-to-metal and ligand charge transfer (MM′LCT) character. In order to assist further in the assignment of the most dominant electronic transitions appearing in the simulated spectra, we calculated the % charge transfer (%*CT*) character using the following equation [40]:

$$\%CT=\sum_{i,j}\;[C{\left(i\to j\right)}^{2}\left(\%M_{i}-\%M_{j}\right)$$

where *C*(*i* → *j*) is the coefficient of *i* → *j* excitation, and %*M* is the percentage of metal orbital contribution. A positive %*CT* value indicates a net MLCT character, while negative values indicate net LMCT character. The %*CT* values are given in Table S2, along with assignment of the major electronic transitions.

Figure 5 also depicts the 3D representation of the hole (*h*) and electron (*e*) localization corresponding to relevant electronic transitions. Perusal of Figure 5 reveals that the h/e localizations confirm the MO-based assignment of the most important bands in the simulated absorption spectrum of [(Salen)Ru(NO)(SH)@Au_20_].

Upon replacing the salen equatorial ligand with the bpb ligand, no significant changes could be observed in the simulated absorption spectrum (Figure 4, green line). Thus, the high-energy band at 485 nm is retained, while the low-energy band is blue-shifted by about 15 nm, peaking around 610 nm. The high-energy band in the simulated absorption spectrum of [(bpb)Ru(NO)(SH)@Au_20_] comprises a multitude of electronic transitions, with the dominant one found at 488 nm. The latter arises from a combination of electronic excitations, i.e., H–4 → L + 3 (42%), H–3 → L + 2 (14%) and HOMO → L + 5 (10%). The 3D surfaces of all MOs involved in these electronic excitations are depicted in Figure S2. Accordingly, H–4 and H–3 are located mainly on the Au_20_ NP, while the HOMO is located mainly on the bpb ligand. On the other hand, all unoccupied MOs involved in these excitations—namely, L + 2, L + 3, and L + 5—are also located on the Au_20_ NP. Therefore, the electronic transition at 488 nm—and, by extension, the high-energy band—could be assigned as MC, similar to the respective band found in the simulated absorption spectrum of [(Salen)Ru(NO)(SH)@Au_20_]. On the other hand, the low-energy band peaking around 610 nm comprises two electronic transitions, with the dominant one found at 612 nm. The latter is due to a combination of electronic excitations, i.e., H–5 → L + 1 (36%) and HOMO → L + 1 (55%). H–5 is mainly located on the Au_20_ NP (see Figure S2), while L + 1 is located on the Ru-NO framework. Taking into account that the HOMO is located on the bpb ligand, we could assign the electronic transition at 612 nm—and, by extension, the low-energy band—as having a complex MM’LCT/LM’L’CT character. Thus, upon changing the equatorial salen ligand with the bpb ligand, the character of the low-energy band is altered.

Next, the simulated absorption spectrum of [(Porph)Ru(NO)(SH)@Au_20_] (red line, Figure 3) also exhibits two absorption bands, being very similar to those calculated for the salen and bpb hybrid Ru complexes. However, with respect to the latter, both bands are red-shifted by 15–25 nm, with the high-energy band appearing around 500 nm and the low-energy band around 650 nm. The high-energy band comprises a multitude of electronic transitions, with the most dominant one found at 491 nm. This band is due to a combination of excitations, namely, H–4 → L + 4 (16%), H-3 → L + 3 (24%), and H–3 → L + 5 (20%). Both H–4 and H–3 are located on the Au_20_ NP, while L + 3, L + 4, and L + 5 are located on both Au_20_ NP as well as on the porphyrin and NO ligands. Thus, the transition at 491 nm—and, by extension, the respective band around 500 nm—could be assigned a complex MC/MLCT character. On the other hand, the low-energy band comprises a group of electronic transitions, with the most dominant one found at 634 nm. The latter arises from the H–1 → L + 1 (30%), HOMO → L + 1 (41%) excitations combination. H–1 is localized on the Au_20_ NP, and the HOMO is on both Au_20_ NP and the thiol ligand, while L + 1 is located on the Ru-NO framework (Figure S3); thus, the band around 650 nm could be described as MLCT/LL’CT/MM’CT.

Finally, the simulated absorption spectrum of [(Pc)Ru(NO)(SH)@Au_20_] (black line, Figure 3)—although being qualitatively similar to those observed for the rest of the Ru complexes under study, exhibiting two bands—also features some striking differences as compared to them. Thus, a significant red-shift is observed for both the high- and low-energy bands, ranging from 80 nm up to 95 nm for the former, and from 170 up to 210 nm for the latter. Another remarkable difference is that the high-energy band for the Ru complex bearing the phthalocyanine ligand is a 7–8-fold increase in its intensity as compared to any of the other Ru complexes under study bearing the salen, bpb, or porphyrin ligands. The high-energy band for the phthalocyanine Ru system peaks around 580 nm (Figure 3) and comprises a large group of electronic transitions. The most dominant one is at 582 nm, arising mainly from an H-1 → L + 3 (80%) excitation. H-1 is located on the Pc equatorial ligand, while L + 3 is located on both RU-NO framework as well as on the Pc ligand. Thus, the transition at 582 nm—and, by extension, the band around 580 nm—could be assigned as LMCT/LL’CT/IL. In addition, the low-energy band, peaking around 820 nm, within NIR, comprises four electronic transitions, with the most dominant amongst them found at 793 nm. The latter arises from a HOMO → L + 1 (89%) excitation, where the HOMO is located on the Au_20_ NP and the thiol ligand, while L + 1 is located on the Ru-NO framework and the Pc ligand (Figure S4). Therefore, the band exhibited by the [(Pc)Ru(NO)(SH)@Au_20_] complex, within the NIR region, could be assigned as MLCT/MM’CT/LL’CT.

#### 2.3.2. Emission

**Scheme 1:**
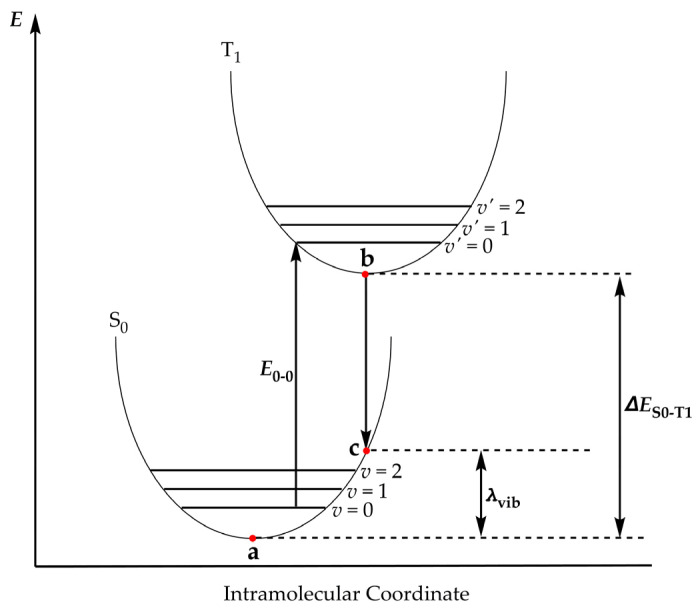
Photophysical parameters relevant to emission.

Next, we will delve into the emission properties of the Ru complexes under investigation. Along these lines, we have calculated a series of photophysical parameters related to emission. These parameters are (a) the vibrational reorganization energy, *λ*_vib_, derived from the energy difference between the S_0_ state and the Franck—Condon S_0_ state; and (b) the zero–zero transition energy, *E*_0-0_, calculated as the Zero-Point Energy (ZPE)-corrected total electronic energies of the T_1_ and S_0_ states and (c) the total electronic energy difference between the T_1_ and S_0_ states. The values of the parameters related to the emission properties of the hybrid complexes under study are tabulated in Table 3. The emission parameters are also depicted in Scheme 1 to clarify their meaning.

Perusal of Table 3 reveals that the vibrational reorganization energies (*λ*_vib_) of 0.76–0.87 eV observed across the series point to pronounced structural relaxation between the singlet and triplet manifolds. This substantial reorganization is consistent with the flexible coordination environment of Ru–NO bound to Au_20_ and the involvement of charge-transfer states. The consequence is a broad and red-shifted emission profile that naturally falls into the near-infrared region, a highly favorable feature for bioimaging applications, where tissue penetration and low background are critical.

Next, the calculated zero–zero transition energies (*E*_0-0_) and the total electronic energy difference between the T_1_ and S_0_ states (Δ*E*_S0-T1_) translate to emission wavelengths in the 1800–3800 nm range, confirming that the lowest-energy radiative processes in these Ru–Au_20_ hybrids occur deep in the near-infrared range. Such long-wavelength transitions arise from the stabilization of triplet states through extensive metal–cluster–ligand delocalization and charge-transfer contributions. Emission in this spectral region overlaps with the NIR-II biological window, which is highly advantageous for in vivo theranostics, providing superior tissue penetration and minimal autofluorescence compared to visible emitters. These zero–zero energies thus reinforce the conclusion that the Ru–Au_20_ architectures are intrinsically predisposed to function as NIR-active platforms for combined imaging and therapeutic applications.

## 3. Computational Details

Full geometric optimization of the Ru (II) complexes under study was performed without symmetry constraints, using the Gaussian16, Rev. C.01 software [41]. The PBE0 functional of Perdew, Burke, and Ernzerhof was employed throughout the calculations [42,43,44,45,46,47]. The effective core potential, LANL2DZ basis set [48,49,50,51], was used for the Ru metal center, while for all non-metal atoms we employed the 6-31G(d,p) basis set as implemented in the Gaussian16, Rev. C.01 software. The combination of the PBE0 functional along with LANL2DZ and 6-31G(d,p) basis sets for heavy and light atoms, respectively, offers an appropriate balance between accuracy and feasibility, keeping the computational cost low for the relatively large Ru–Au_20_ hybrid systems. Local minima were verified upon calculating the vibrational modes, resulting in zero imaginary frequencies (NImag = 0). The computational method is abbreviated as PBE0/LanL2DZ(Ru)U6-31G(d,p)(E). Water solvent effects were taken into account, employing the Polarizable Continuum Model (PCM) using the integral equation formalism variant (IEF-PCM), as implemented in the Gaussian16, Rev. C.01 software [52]. The Natural Bond Orbital (NBO) method of Weinhold et al. was used [53]. Dispersion corrections (D3/D4) were not included, because the NEDA analysis indicated that the Ru–S–Au interaction is dominated by electrostatic and charge-transfer terms, and our benchmark tests already reproduced key structural and spectroscopic observables; dispersion effects are expected to play only a minor role in these strongly bonded intramolecular systems. Time-dependent density functional theory (TD-DFT) calculations [54,55,56,57] were carried out upon taking into account the 30 least excited states. Multiwfn 3.8 [58] and GaussSum 2.2.6.1 [59] wave-function analyzers were also used to visualize the results. The emission-related parameters were obtained following the procedure illustrated in Scheme 1. Specifically, *E*_0–0_ was computed as the vertical T_1_ → S_0_ emission energy at the optimized T_1_ geometry, Δ*E*_T1–S0_ corresponds to the adiabatic energy difference between the optimized triplet and ground-state minima, and the vibrational reorganization energy *λ*_vib_ was calculated as the difference between the vertical S_0_ → T_1_ excitation at the T_1_ geometry and the adiabatic T_1_−S_0_ energy gap. All quantities were obtained at the same PBE0/LANL2DZ–6-31G(d,p) level used for the geometric optimizations and TDDFT calculations. Finally, wave-function stability was examined using SCF stability analysis (*Stable = Opt keyword in G16*), employing our computational method. RKS/UKS solutions were confirmed to be stable.

## 4. Conclusions

This theoretical investigation elucidates the structural, electronic, and photophysical features governing the behavior of hybrid Ru–NO/Au_20_ systems relevant to photoresponsive theranostics. The Ru–NO fragment retains a linear geometry typical of {RuNO}^6^ species but exhibits clear signatures of metal-to-ligand π-backbonding, reducing the N–O bond order and enabling efficient photoactivation. The Au_20_ nanocluster acts as an electronically active support that mediates long-range charge transfer through delocalized Ru–S–Au linkages. NBO, NEDA, and ETS–NOCV analyses consistently show that the bonding framework is dominated by charge-transfer and polarization interactions, confirming a strong donor–acceptor coupling between the Ru chromophore and the gold core. TDDFT-derived absorption spectra display visible and NIR charge-transfer bands that evolve systematically with the equatorial ligand environment. The calculated zero–zero transition energies and reorganization energies point to deep NIR-II emission, suggesting that these Ru–Au_20_ assemblies could serve as luminescent sensors and NO-releasing agents under biologically transparent conditions. Among the systems studied, the phthalocyanine hybrid [(Pc)Ru(NO)(SH)@Au_20_] displays the most favorable combination of strong visible absorption, lowest reorganization energy, and deepest NIR-II emission, identifying it as the most promising candidate for luminescence-based NO detection. Overall, the results demonstrate that coupling molecular Ru–NO photoNORMs to plasmonic nanoclusters offers a rational route toward multifunctional, luminescent theranostic architectures, providing a theoretical foundation for future experimental realization and biomedical application.

## Figures and Tables

**Table 1:** Natural charges (Q) and Wiberg bond indices (WBIs) derived from the NBO analysis on the [(L)Ru(NO)(SH)@Au_20_] complexes at the PBE0/LanL2DZ(Ru)U6-31G(d,p)(E) level in water solvent.

Parameter	[(L)Ru(NO)(SH)@Au_20_]			
	L = Salen	L = bpb	L = porph	L = pc
*Q*_Ru_	0.695	0.582	0.597	0.552
*Q*_N_	0.363	0.338	0.400	0.388
*Q*_S_	−0.474	−0.446	−0.452	−0.441
*Q*_Au_	0.065	0.061	0.083	0.059
WBI(Ru-NO)	1.242	1.216	1.252	1.204
WBI(N-O)	1.948	1.926	1.965	1.980
WBI(Ru-S)	0.485	0.481	0.479	0.493
WBI(S-Au)	0.272	0.265	0.266	0.246

**Table 2:** NEDA energy components for selected bonds in the [(L)Ru(NO)(SH)@Au_20_] hybrid complexes. All values are given in kcal mol^−1 1^.

L	Bond	ES	POL	CT	XC	Δ*E*
	Ru-N	−75.1	−212.0	−520.5	−75.5	−201.0
[(salen)Ru(NO)(SH)@Au_20_]	Ru-S	−119.6	−130.7	−161.6	−67.6	−96.7
	Au-S	−74.4	−45.1	−85.7	−56.3	−28.3
	Ru-N	−95.7	−187.5	−525.2	−76.5	−202.0
[(bpb)Ru(NO)(SH)@Au_20_]	Ru-S	−127.0	−128.0	−165.3	−66.9	−102.2
	Au-S	−73.2	−44.0	−82.5	−54.2	−27.6
	Ru-N	−79.2	−220.7	−521.7	−78.2	−195.8
[(porph)Ru(NO)(SH)@Au_20_]	Ru-S	−115.5	−125.8	−162.2	−67.7	−88.5
	Au-S	−74.4	−47.9	−85.1	−56.9	−27.4
	Ru-N	−66.2	−218.5	−500.3	−75.9	−180.8
[(pc)Ru(NO)(SH)@Au_20_]	Ru-S	−119.1	−139.6	−184.9	−75.2	−93.5
	Au-S	−90.8	−56.3	−90.8	−61.8	−22.6

^1^ The core (XC + deformation/self-exchange) component is not listed separately but is included in the reported Δ*E* values.

**Table 3:** Selected photophysical parameters of the [(L)Ru(NO)(SH)@Au_20_] complexes calculated at the PBE0/LanL2DZ(Ru)U6-31G(d,p)(E) level in water solvent.

Complex	*λ*_vib_ (eV)	*E*_0-0_ (nm)	Δ*E*_S0-T1_ (nm)
[(salen)Ru(NO)(SH)@Au_20_]	0.790	1931	1733
[(bpb)Ru(NO)(SH)@Au_20_]	0.837	1803	1643
[(porph)Ru(NO)(SH)@Au_20_]	0.866	2232	1972
[(pc)Ru(NO)(SH)@Au_20_]	0.760	3737	3010

## Data Availability

The data presented in this study are available on request from the corresponding author. The data are not publicly available due to ongoing research and the inclusion of unpublished experimental results.

## Supplementary Materials

The following supporting information can be downloaded at https://www.mdpi.com/article/10.3390/molecules30224432/s1: Figure S1: Simulated IR spectra calculated at the PBE0/LanL2DZ(Ru)U6-31G(d,p)(E) level of theory, in water solvent. Figure S2: The 3D surfaces of MOs involved in the electronic excitations relevant to the most important electronic transitions in the simulated absorption spectrum of [(bpb)Ru(NO)(SH)@Au_20_] in water solvent. Figure S3: The 3D surfaces of MOs involved in the electronic excitations relevant to the most important electronic transitions in the simulated absorption spectrum of [(porph)Ru(NO)(SH)@Au_20_] in water solvent. Figure S4: The 3D surfaces of MOs involved in the electronic excitations relevant to the most important electronic transitions in the simulated absorption spectrum of [(pc)Ru(NO)(SH)@Au_20_] in water solvent. Figure S5. Absorption spectra of (a) [(CN)_5_Ru(NO)]^2−^ and (b) [(NH_3_)_5_Ru(NO)]^3+^ complexes calculated at PBE0/LanL2DZ(Ru)U6-31G(d,p)(E) level of theory, in water solvent. Table S1: Cartesian coordinates and energetic data. Table S2: Major electronic transitions and assignment for the [(L)Ru(NO)(SH)@Au_20_] complexes calculated at the PBE0/LanL2DZ(Ru)U6-31G(d,p)(E) level in water solvent. Table S3: %*CT* character of the 30 least excited states of [(Salen)Ru(NO)(SH)@Au_20_]. Table S4: %*CT* character of the 30 least excited states of [(bpb)Ru(NO)(SH)@Au_20_]. Table S5: %*CT* character of the 30 least excited states of [(porph)Ru(NO)(SH)@Au_20_]. Table S6: %*CT* character of the 30 least excited states of [(pc)Ru(NO)(SH)@Au_20_].
