# Supplementary material for: Photoresponsive Ru Complex–Gold Nanoparticle Hybrids for Theranostics: A Theoretical Study of Electronic Structure and Luminescence-Based Detection
Source: Molecules. 2025 Nov 16;30(22):4432. doi: 10.3390/molecules30224432 (PMC12655125; doi:10.3390/molecules30224432)
Supplement: Supplementary file 1 [file molecules-30-04432-s001.zip › molecules-3946863-supplementary.pdf]

Supplementary Material

Photoresponsive Ru Complex – Gold Nanoparticle Hybrids for Theranostics: A Theoretical Study of Electronic Structure and Luminescence Based Detection

Niq Catevas and Athanassios Tsipis*

Photoresponsive Ru Complex – Gold Nanoparticle Hybrids for Theranostics: A Theoretical Study of Electronic Structure and Luminescence Based Detection

Niq Catevas[a] and Athanassios Tsipis*[a]

[a] Prof. Dr. A. Tsipis, PhD student N. Catevas
Department of Chemistry
University of Ioannina
T.Th.1186, Campus Ioannina University, Ioannina, Greece
E-mail: attsipis@uoi.gr

Supplementary Information

Contents

Figure S1. Simulated IR spectra calculated at PBE0/LanL2DZ(Ru)U6-31G(d,p)(E) level of theory, in water solvent.

Figure S2. 3D surfaces of MOs involved into the electronic excitations relevant to the most important electronic transitions in the simulated absorption spectrum of [(bpb)Ru(NO)(SH)@Au_{20}] in water solvent.

Figure S3. 3D surfaces of MOs involved into the electronic excitations relevant to the most important electronic transitions in the simulated absorption spectrum of [(porph)Ru(NO)(SH)@Au_{20}] in water solvent.

Figure S4. 3D surfaces of MOs involved into the electronic excitations relevant to the most important electronic transitions in the simulated absorption spectrum of [(pc)Ru(NO)(SH)@Au_{20}] in water solvent.

Figure S5. Absorption spectra of (a) $[(CN)_5Ru(NO)]^{2-}$ and (b) $[(NH_3)_5Ru(NO)]^{3+}$ complexes calculated at PBE0/LanL2DZ(Ru)U6-31G(d,p)(E) level of theory, in water solvent.

Table S1. Cartesian coordinates and energetic data.

Table S2. Major electronic transitions and assignment for the [(L)Ru(NO)(SH)@Au_{20}] complexes calculated at the PBE0/LanL2DZ(Ru)U6-31G(d,p)(E) level in water solvent.

Table S3. %*CT* character of the lowest 30 excited states of [(Salen)Ru(NO)(SH)@Au_{20}]

Table S4. %*CT* character of the lowest 30 excited states of [(bpb)Ru(NO)(SH)@Au_{20}]

Table S5. %*CT* character of the lowest 30 excited states of [(porph)Ru(NO)(SH)@Au_{20}]

Table S6. %*CT* character of the lowest 30 excited states of [(pc)Ru(NO)(SH)@Au_{20}]

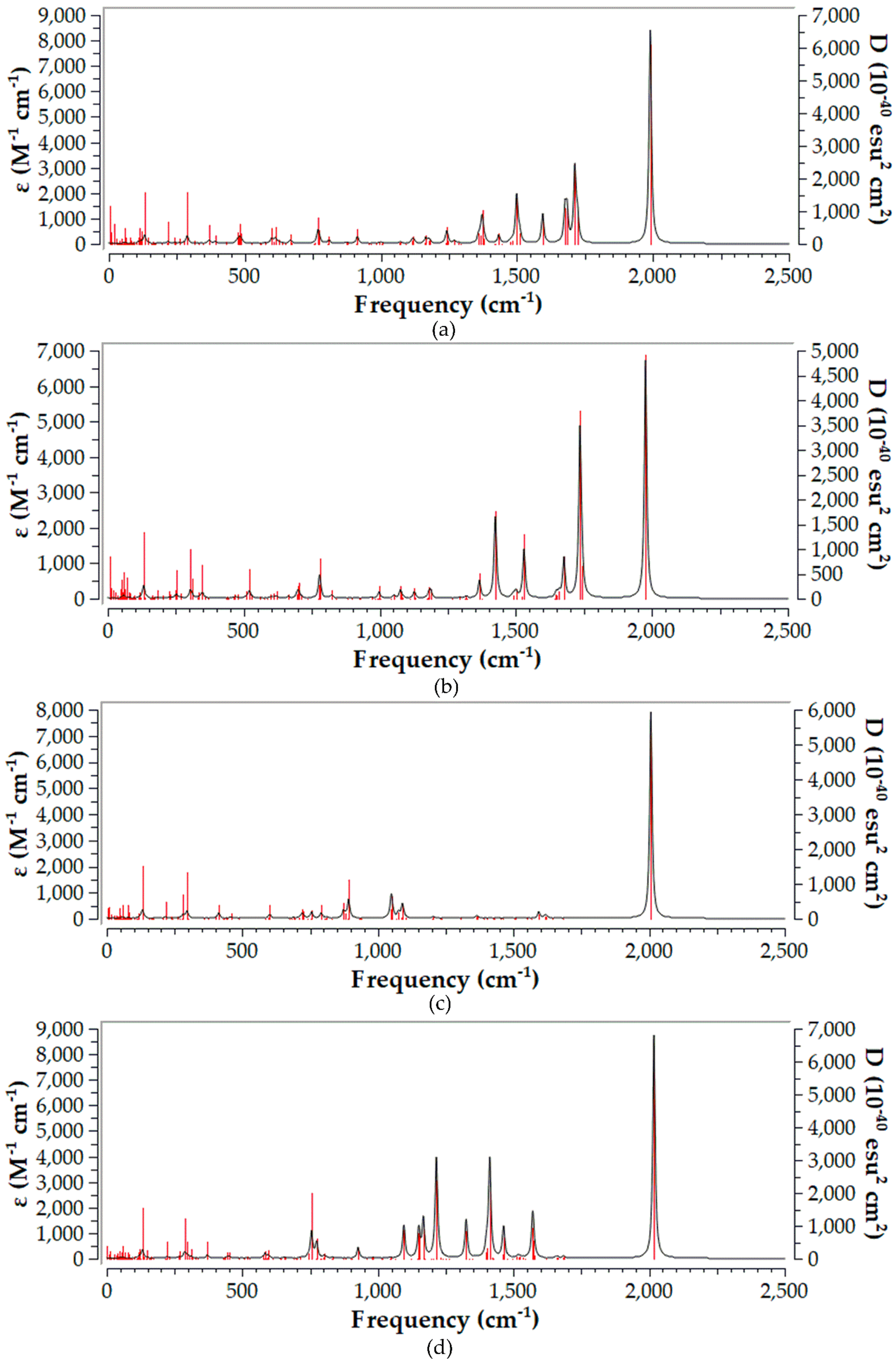

Figure S1. Simulated IR spectra calculated at PBE0/LanL2DZ(Ru)U6-31G(d,p)(E) level of theory, in water solvent for (a) [(Salen)Ru(NO)(SH)], (b) [(bpb)Ru(NO)(SH)], (c) [(Porph)Ru(NO)(SH)] and (d) [(Pc)Ru(NO)(SH)] complexes.

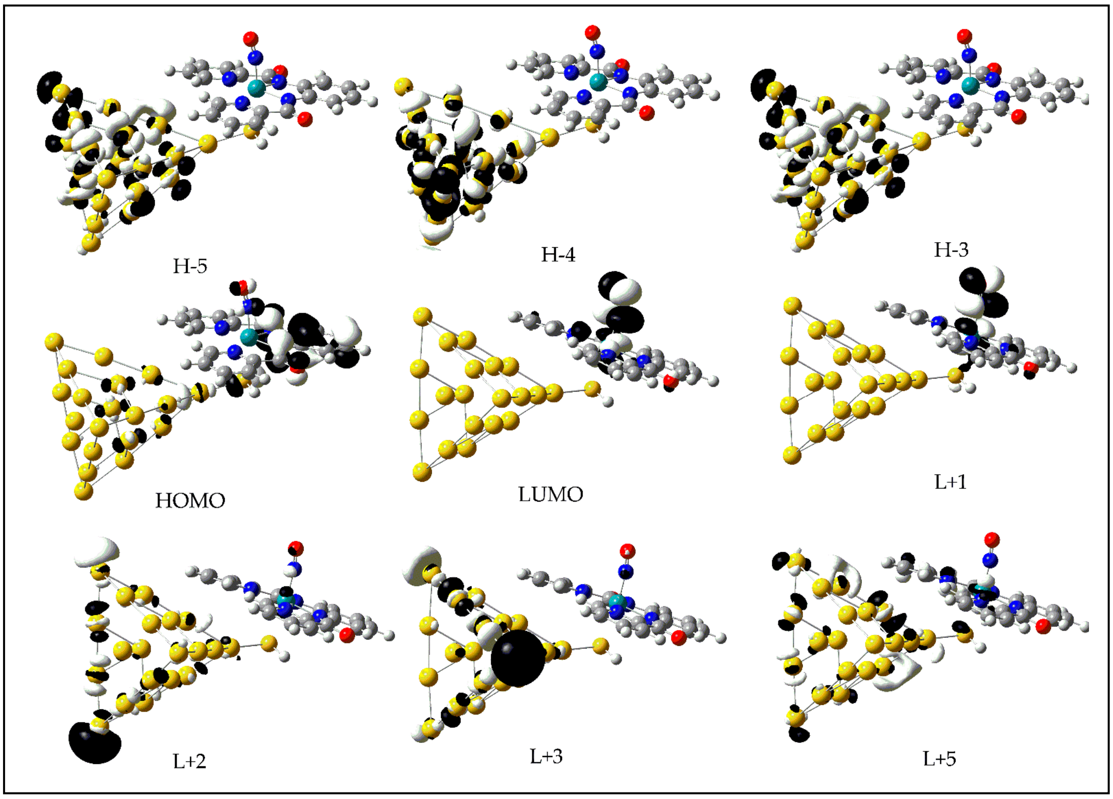

Figure S2. 3D surfaces of MOs involved into the electronic excitations relevant to the most important electronic transitions in the simulated absorption spectrum of [(bpb)Ru(NO)(SH)@Au_{20}] in water solvent.

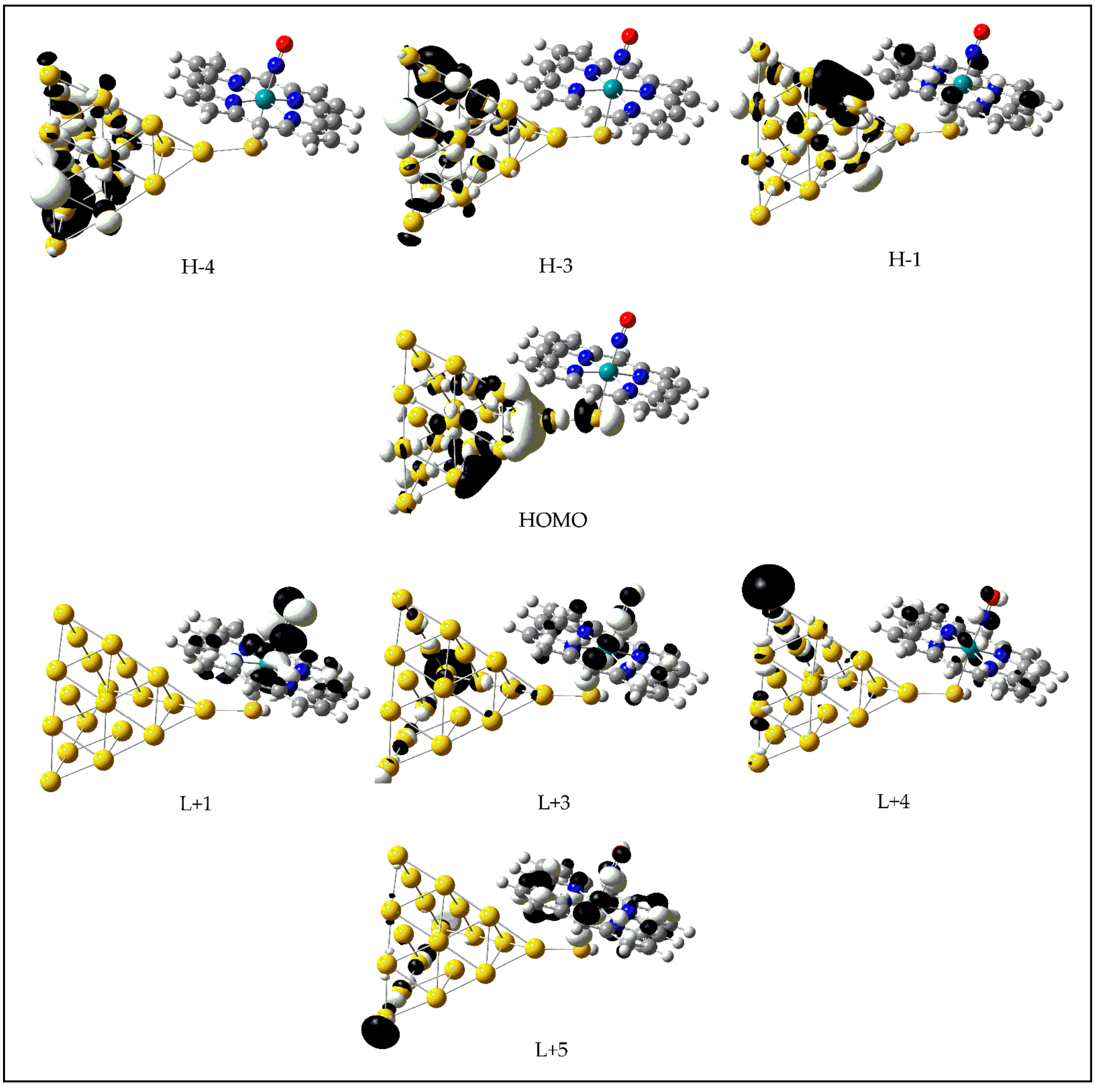

Figure S3. 3D surfaces of MOs involved into the electronic excitations relevant to the most important electronic transitions in the simulated absorption spectrum of [(porph)Ru(NO)(SH)@Au_{20}] in water solvent.

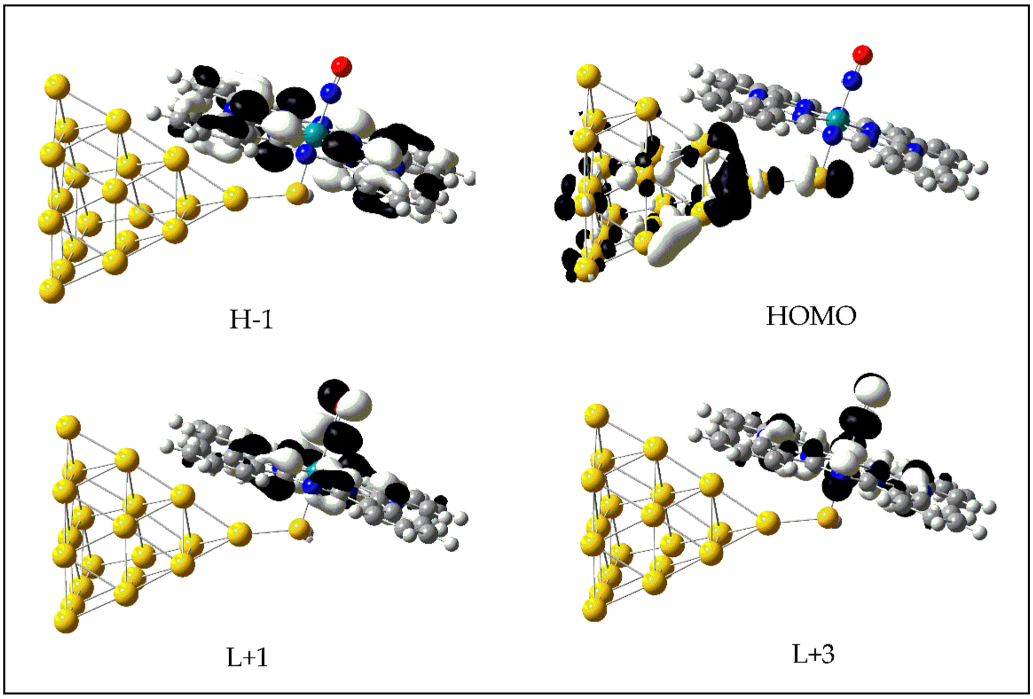

Figure S4. 3D surfaces of MOs involved into the electronic excitations relevant to the most important electronic transitions in the simulated absorption spectrum of [(pc)Ru(NO)(SH)@Au_{20}] in water solvent.

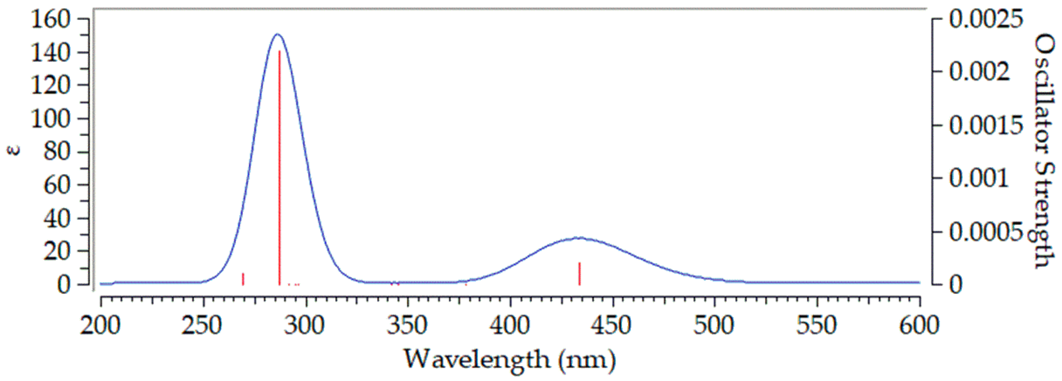

(a)

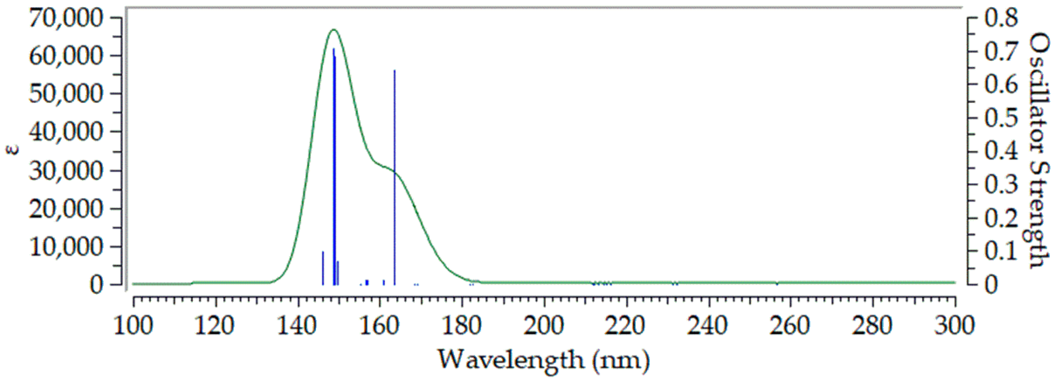

(b)

Figure S5. Absorption spectra of (a) $[(CN)_5Ru(NO)]^{2-}$ and (b) $[(NH_3)_5Ru(NO)]^{3+}$ complexes calculated at PBE0/LanL2DZ(Ru)U6-31G(d,p)(E) level of theory, in water solvent.

Table S1. Cartesian coordinates and energetic data.

[(salen)Ru(NO)(SH)@Au$_{20}$], S$_0$

Ru	-7.542516000	0.398876000	0.303606000
N	-8.564609000	1.019382000	1.602143000
O	-9.315324000	1.407573000	2.372611000
N	-7.517745000	-1.519271000	0.948945000
O	-9.206762000	0.103630000	-0.883490000
C	-9.637925000	-2.162972000	-0.064755000
C	-9.943642000	-0.968270000	-0.805388000
C	-11.159428000	-0.961861000	-1.538535000
C	-12.016678000	-2.040829000	-1.543405000
C	-11.717638000	-3.208266000	-0.818485000
C	-10.543765000	-3.253423000	-0.103476000
C	-8.445330000	-2.382567000	0.699792000
C	-6.262778000	-1.876421000	1.612756000
N	-5.829505000	0.499242000	1.375196000
O	-7.229867000	2.221153000	-0.590314000
C	-5.076661000	2.641900000	0.499741000
C	-6.161352000	2.946426000	-0.389641000
C	-6.065598000	4.152838000	-1.130016000
C	-4.989556000	5.007265000	-1.002799000
C	-3.930782000	4.712218000	-0.127637000
C	-3.988372000	3.544700000	0.601910000
C	-4.975359000	1.462560000	1.313803000
C	-5.684581000	-0.635406000	2.283971000
Au	-3.910710000	-0.469528000	-1.290574000
Au	-1.844449000	1.369613000	-0.906136000
Au	-1.349181000	-1.232303000	-2.036468000
Au	-2.065389000	-1.007391000	0.736086000
Au	0.546394000	-1.875177000	0.003388000
Au	0.776695000	0.656403000	-1.750986000
Au	-0.011143000	0.906729000	1.229866000
Au	0.188061000	3.145168000	-0.502252000
Au	1.194367000	-1.969637000	-2.748986000
Au	-0.250963000	-1.504776000	2.711216000
Au	2.871677000	0.106779000	0.462879000
Au	2.223701000	2.622076000	1.618716000
Au	2.976316000	2.379563000	-1.240680000
Au	2.009186000	0.397687000	3.159291000
Au	3.462620000	-0.067234000	-2.314233000
Au	2.540559000	-2.270017000	1.989530000
Au	3.248432000	-2.491126000	-0.623764000
Au	2.367120000	4.788261000	-0.066270000
Au	1.729420000	-1.926835000	4.594491000
Au	3.845321000	-2.599786000	-3.297702000
H	-11.390094000	-0.062153000	-2.100870000
H	-12.936586000	-1.984342000	-2.119549000
H	-12.394798000	-4.055439000	-0.827572000
H	-10.286758000	-4.148700000	0.457817000
H	-8.309677000	-3.389101000	1.104303000
H	-5.560361000	-2.232761000	0.848031000
H	-6.419314000	-2.677653000	2.341588000
H	-6.884640000	4.383163000	-1.804512000

H	-4.967627000	5.922590000	-1.588441000
H	-3.087053000	5.386981000	-0.026941000
H	-3.180572000	3.295277000	1.286610000
H	-4.079203000	1.379244000	1.935058000
H	-4.632396000	-0.795658000	2.544719000
H	-6.243607000	-0.411097000	3.200865000
S	-6.350949000	-0.514268000	-1.645184000
H	-6.440929000	0.626625000	-2.353803000

Sum of electronic and zero-point Energies= -4208.697617
Sum of electronic and thermal Energies= -4208.629902
Sum of electronic and thermal Enthalpies= -4208.628958
Sum of electronic and thermal Free Energies= -4208.832190

[(salen)Ru(NO)(SH)@Au_{20}], T_1

Ru	-7.387733000	0.363691000	0.242304000
N	-8.458481000	1.141577000	1.555618000
O	-9.365404000	1.898620000	1.580883000
N	-7.524480000	-1.508695000	0.988320000
O	-9.094191000	0.124291000	-0.932176000
C	-9.676857000	-2.062940000	-0.003006000
C	-9.899446000	-0.884439000	-0.805205000
C	-11.119803000	-0.840974000	-1.539199000
C	-12.049058000	-1.857102000	-1.493860000
C	-11.825877000	-3.004629000	-0.712288000
C	-10.654026000	-3.088060000	0.006310000
C	-8.499467000	-2.327350000	0.782373000
C	-6.311124000	-1.907582000	1.698950000
N	-5.744026000	0.432994000	1.410109000
O	-6.974269000	2.212129000	-0.626243000
C	-4.862085000	2.538158000	0.565658000
C	-5.891323000	2.883675000	-0.384311000
C	-5.691844000	4.086400000	-1.122562000
C	-4.593793000	4.900055000	-0.938176000
C	-3.601796000	4.568626000	0.000493000
C	-3.750894000	3.403764000	0.726789000
C	-4.852206000	1.362420000	1.402076000
C	-5.703048000	-0.674602000	2.360288000
Au	-3.832006000	-0.473546000	-1.392654000
Au	-1.854927000	1.436679000	-0.852888000
Au	-1.268849000	-1.154415000	-2.185326000
Au	-2.057112000	-1.118880000	0.663907000
Au	0.528478000	-1.902325000	-0.129169000
Au	0.739873000	0.770899000	-1.699382000
Au	-0.085480000	0.811262000	1.269740000
Au	0.169575000	3.184225000	-0.298086000
Au	1.304641000	-1.788389000	-2.870406000
Au	-0.257449000	-1.703608000	2.618144000
Au	2.760399000	0.095587000	0.485556000
Au	2.107638000	2.531547000	1.826017000
Au	2.922999000	2.486927000	-1.052830000
Au	1.906684000	0.206353000	3.204556000
Au	3.444868000	0.131594000	-2.284043000
Au	2.500941000	-2.393676000	1.854610000
Au	3.242242000	-2.435735000	-0.753382000

Au	2.314767000	4.807466000	0.294830000
Au	1.704073000	-2.223923000	4.480529000
Au	3.953040000	-2.329108000	-3.408567000
H	-11.291690000	0.043281000	-2.146706000
H	-12.964708000	-1.767346000	-2.073481000
H	-12.556176000	-3.806382000	-0.679686000
H	-10.456798000	-3.970679000	0.611670000
H	-8.435937000	-3.321922000	1.234780000
H	-5.598661000	-2.308149000	0.964826000
H	-6.517568000	-2.687910000	2.439673000
H	-6.462746000	4.348844000	-1.841727000
H	-4.502798000	5.812292000	-1.522844000
H	-2.739272000	5.209536000	0.154116000
H	-3.000088000	3.134953000	1.467941000
H	-4.001008000	1.265023000	2.083706000
H	-4.679386000	-0.877866000	2.696812000
H	-6.307414000	-0.392489000	3.232083000
S	-6.186013000	-0.639299000	-1.738324000
H	-6.300854000	0.455406000	-2.514733000

Sum of electronic and zero-point Energies= -4208.674014
Sum of electronic and thermal Energies= -4208.606670
Sum of electronic and thermal Enthalpies= -4208.605726
Sum of electronic and thermal Free Energies= -4208.806764

[(bpb)Ru(NO)(SH)@Au_{20}], S_0

Ru	-7.379438000	-0.016916000	0.532695000
O	-9.199519000	-3.459513000	-0.775351000
O	-8.767878000	3.673296000	-0.627432000
O	-8.097898000	0.057526000	3.345217000
N	-8.738121000	-1.246661000	-0.255726000
N	-8.552266000	1.394730000	-0.250407000
N	-6.413636000	-1.913382000	0.838811000
N	-6.181100000	1.738549000	0.900465000
C	-9.823508000	-0.555869000	-0.817139000
C	-9.725834000	0.865043000	-0.811835000
C	-10.762102000	1.630153000	-1.355830000
C	-11.881266000	1.001693000	-1.896433000
C	-11.976415000	-0.387194000	-1.902717000
C	-10.954211000	-1.167197000	-1.367674000
C	-8.491470000	-2.572268000	-0.295946000
C	-7.168498000	-2.917303000	0.341527000
C	-6.745851000	-4.237598000	0.401116000
C	-5.521329000	-4.530889000	0.992490000
C	-4.756094000	-3.490662000	1.508691000
C	-5.238941000	-2.190285000	1.411726000
C	-8.163210000	2.686355000	-0.204564000
C	-6.813297000	2.844835000	0.451177000
C	-6.244174000	4.104551000	0.572476000
C	-4.997725000	4.229364000	1.177437000
C	-4.356272000	3.085236000	1.638891000
C	-4.982656000	1.853097000	1.478661000
Au	-3.818922000	-0.112416000	-1.237169000
Au	-1.777540000	1.538329000	-0.303635000
Au	-1.261265000	-0.486303000	-2.278548000

Au	-1.882896000	-1.253207000	0.418259000
Au	0.717407000	-1.733205000	-0.633409000
Au	0.835173000	1.247670000	-1.403286000
Au	0.136280000	0.437299000	1.503411000
Au	0.225442000	3.136508000	0.632012000
Au	1.268325000	-0.846415000	-3.268151000
Au	0.001252000	-2.347826000	2.056629000
Au	3.009548000	0.044966000	0.430845000
Au	2.343660000	1.988257000	2.393847000
Au	3.009237000	2.768203000	-0.387516000
Au	2.234917000	-0.637738000	3.081210000
Au	3.514778000	0.854080000	-2.248558000
Au	2.781903000	-2.721951000	1.045611000
Au	3.405975000	-2.006515000	-1.499415000
Au	2.376791000	4.603875000	1.556997000
Au	2.054421000	-3.324940000	3.625792000
Au	3.908971000	-1.169266000	-4.061542000
H	-10.670689000	2.708395000	-1.347681000
H	-12.680849000	1.606522000	-2.313952000
H	-12.850759000	-0.873506000	-2.325226000
H	-11.010679000	-2.247876000	-1.369651000
H	-7.391061000	-5.000766000	-0.018736000
H	-5.170622000	-5.556109000	1.052193000
H	-3.794906000	-3.666194000	1.979878000
H	-4.673536000	-1.351884000	1.801323000
H	-6.797426000	4.954986000	0.190804000
H	-4.534402000	5.204527000	1.287899000
H	-3.383380000	3.130011000	2.116311000
H	-4.512238000	0.937683000	1.817856000
N	-7.944000000	0.023343000	2.210159000
S	-6.256504000	-0.142056000	-1.644772000
H	-6.379877000	1.145785000	-2.015567000

Sum of electronic and zero-point Energies= -4393.058163

Sum of electronic and thermal Energies= -4392.988324

Sum of electronic and thermal Enthalpies= -4392.987380

Sum of electronic and thermal Free Energies= -4393.194308

[(bpb)Ru(NO)(SH)@Au_{20}], T_1

Ru	7.123145000	-0.050715000	0.457153000
O	8.150561000	1.026990000	2.890658000
Au	3.698340000	-0.415989000	-1.489204000
Au	1.115240000	-0.882370000	-2.357882000
Au	1.778596000	1.457084000	-0.663589000
Au	1.880396000	-1.288140000	0.445985000
Au	0.022716000	0.629058000	1.398485000
Au	-0.830438000	1.050722000	-1.581037000
Au	-0.727161000	-1.820325000	-0.410226000
Au	-1.498237000	-1.274461000	-3.104398000
Au	-0.135638000	3.200683000	0.168930000
Au	0.074722000	-2.071094000	2.328032000
Au	-2.870035000	0.143959000	0.493582000
Au	-3.448640000	-2.153731000	-1.104890000
Au	-3.553430000	0.626866000	-2.235160000
Au	-2.708973000	-2.533316000	1.479632000

Au	-2.922296000	2.749508000	-0.667382000
Au	-2.022417000	-0.192897000	3.193443000
Au	-2.125943000	2.318435000	2.176839000
Au	-4.156201000	-1.621382000	-3.713110000
Au	-1.908279000	-2.788548000	4.099148000
Au	-2.201992000	4.808362000	1.014214000
N	7.812753000	0.151042000	2.169315000
S	6.058101000	-0.248600000	-1.825063000
O	9.257136000	-3.403190000	-0.686645000
O	8.415078000	3.744596000	-0.536321000
N	8.600893000	-1.209610000	-0.271596000
N	8.267724000	1.436877000	-0.257708000
N	6.342988000	-2.024969000	0.876431000
N	5.865844000	1.642591000	0.846972000
C	9.652780000	-0.451866000	-0.803160000
C	9.477299000	0.968464000	-0.789899000
C	10.489490000	1.788194000	-1.303391000
C	11.655957000	1.228929000	-1.820947000
C	11.825960000	-0.152896000	-1.835967000
C	10.832144000	-0.989699000	-1.332577000
C	8.474767000	-2.544834000	-0.252713000
C	7.181354000	-2.975714000	0.404013000
C	6.882961000	-4.328870000	0.512298000
C	5.698018000	-4.719193000	1.126810000
C	4.845900000	-3.737198000	1.621346000
C	5.209488000	-2.402348000	1.474886000
C	7.842621000	2.706683000	-0.172054000
C	6.470415000	2.788353000	0.456748000
C	5.857425000	4.025933000	0.615474000
C	4.595947000	4.092581000	1.195494000
C	3.980121000	2.910517000	1.596184000
C	4.651304000	1.706789000	1.401684000
H	6.057300000	1.049673000	-2.186876000
H	10.336637000	2.859961000	-1.287704000
H	12.431566000	1.880514000	-2.213810000
H	12.735503000	-0.588225000	-2.240631000
H	10.944690000	-2.066562000	-1.340050000
H	7.595090000	-5.040730000	0.110388000
H	5.445909000	-5.770616000	1.222986000
H	3.912543000	-3.986713000	2.114827000
H	4.574383000	-1.606562000	1.850420000
H	6.394683000	4.905739000	0.279713000
H	4.101123000	5.048790000	1.334763000
H	2.997103000	2.907015000	2.055210000
H	4.202070000	0.763668000	1.694638000

Sum of electronic and zero-point Energies= -4393.032893
Sum of electronic and thermal Energies= -4392.962546
Sum of electronic and thermal Enthalpies= -4392.961602
Sum of electronic and thermal Free Energies= -4393.171155

[(porph)Ru(NO)(SH)@Au_{20}], S_0

Ru	7.549519000	-0.005706000	0.326681000
O	9.290511000	0.080717000	2.642828000
C	7.039140000	3.390684000	0.463637000

C	10.262208000	0.489306000	-1.715218000
C	7.843127000	-3.400374000	-0.120315000
C	4.719459000	-0.500332000	2.204095000
C	5.071038000	0.824106000	1.938763000
C	4.358378000	1.984272000	2.408790000
C	4.999267000	3.076381000	1.906132000
C	6.114898000	2.593347000	1.132770000
N	6.123110000	1.227778000	1.172586000
C	8.132653000	2.952609000	-0.280445000
C	9.069325000	3.801368000	-0.970128000
C	9.976840000	2.986851000	-1.576590000
C	9.602967000	1.631272000	-1.267454000
N	8.485947000	1.649508000	-0.482382000
C	9.900788000	-0.832366000	-1.469184000
C	10.570872000	-1.992771000	-1.998322000
C	9.888860000	-3.084770000	-1.555958000
C	8.797046000	-2.603108000	-0.748634000
N	8.840259000	-1.240741000	-0.713636000
C	6.767061000	-2.961468000	0.645636000
C	5.805338000	-3.811418000	1.298604000
C	4.937413000	-2.997932000	1.962697000
C	5.354927000	-1.642219000	1.713680000
N	6.458658000	-1.656803000	0.913194000
H	6.893786000	4.464256000	0.528964000
H	11.136963000	0.646254000	-2.338158000
H	7.948486000	-4.473683000	-0.241970000
H	3.877513000	-0.658497000	2.872873000
H	3.475847000	1.951609000	3.034872000
H	4.752311000	4.120917000	2.044586000
H	9.025800000	4.882494000	-0.982874000
H	10.825485000	3.266755000	-2.186836000
H	11.446321000	-1.961052000	-2.633593000
H	10.093122000	-4.128638000	-1.754852000
H	5.808199000	-4.892565000	1.252425000
H	4.082406000	-3.275943000	2.565681000
Au	3.829952000	-0.097557000	-1.333879000
Au	1.314127000	-1.024744000	-2.066506000
Au	1.646342000	1.597275000	-0.960230000
Au	2.044716000	-0.742008000	0.708703000
Au	-0.134703000	1.031787000	1.191389000
Au	-0.942152000	0.708125000	-1.789986000
Au	-0.502449000	-1.778361000	0.000969000
Au	-1.173717000	-1.947106000	-2.754559000
Au	-0.499488000	3.235297000	-0.563354000
Au	0.270509000	-1.340187000	2.699071000
Au	-2.964565000	0.039716000	0.461313000
Au	-3.154333000	-2.590433000	-0.608104000
Au	-3.582462000	-0.203614000	-2.301649000
Au	-2.463538000	-2.295969000	2.000364000
Au	-3.249746000	2.281428000	-1.255589000
Au	-2.104935000	0.413928000	3.145801000
Au	-2.469101000	2.605168000	1.587416000
Au	-3.782758000	-2.761070000	-3.272375000

Au	-1.668060000	-1.876596000	4.598452000
Au	-2.777965000	4.736770000	-0.116017000
N	8.578353000	0.020262000	1.753592000
S	6.245912000	-0.025493000	-1.769895000
H	6.304264000	1.294706000	-2.025371000

Sum of electronic and zero-point Energies= -4318.869696
Sum of electronic and thermal Energies= -4318.802562
Sum of electronic and thermal Enthalpies= -4318.801618
Sum of electronic and thermal Free Energies= -4319.001116

[(porph)Ru(NO)(SH)@Au_{20}], T_1

Ru	7.163045000	-0.018956000	0.291206000
O	8.366619000	0.989354000	2.681100000
C	6.677052000	3.373318000	0.380811000
C	10.112801000	0.438441000	-1.379956000
C	7.590668000	-3.423724000	0.110290000
C	4.191724000	-0.490342000	1.964714000
C	4.571912000	0.847463000	1.691369000
C	3.838217000	2.017684000	2.119951000
C	4.521098000	3.100492000	1.656428000
C	5.681343000	2.596913000	0.958916000
N	5.667152000	1.226301000	0.994777000
C	7.849248000	2.923397000	-0.239670000
C	8.858084000	3.768386000	-0.831010000
C	9.824193000	2.943908000	-1.323059000
C	9.410249000	1.592878000	-1.032997000
N	8.213639000	1.620077000	-0.376994000
C	9.744178000	-0.883851000	-1.133034000
C	10.492294000	-2.052147000	-1.531295000
C	9.781084000	-3.136476000	-1.116072000
C	8.595619000	-2.633925000	-0.462628000
N	8.608749000	-1.275151000	-0.486269000
C	6.432550000	-2.985398000	0.739639000
C	5.451020000	-3.832001000	1.378032000
C	4.511919000	-3.009388000	1.920245000
C	4.908896000	-1.655648000	1.596809000
N	6.060677000	-1.674500000	0.889021000
H	6.540455000	4.449858000	0.428989000
H	11.049896000	0.586717000	-1.909149000
H	7.740002000	-4.498755000	0.066469000
H	3.459652000	-0.617499000	2.761644000
H	2.916331000	1.994167000	2.687831000
H	4.278990000	4.148331000	1.779904000
H	8.819844000	4.849613000	-0.862226000
H	10.736825000	3.214351000	-1.838452000
H	11.436441000	-2.032384000	-2.060328000
H	10.024699000	-4.184188000	-1.236640000
H	5.493014000	-4.913114000	1.410547000
H	3.623883000	-3.275178000	2.480333000
Au	3.771849000	-0.300877000	-1.627635000
Au	1.252742000	-1.246831000	-2.131343000
Au	1.653264000	1.480839000	-1.206494000
Au	2.123504000	-0.804021000	0.635433000
Au	-0.000883000	1.075219000	1.085807000

Au	-0.921399000	0.592765000	-1.811943000
Au	-0.446883000	-1.768719000	0.103052000
Au	-1.317753000	-2.134348000	-2.585140000
Au	-0.409320000	3.204626000	-0.752328000
Au	0.365706000	-1.203751000	2.734085000
Au	-2.867299000	0.113770000	0.521599000
Au	-3.153664000	-2.576840000	-0.353480000
Au	-3.564133000	-0.272961000	-2.223934000
Au	-2.387784000	-2.130213000	2.195341000
Au	-3.159178000	2.262209000	-1.335828000
Au	-1.923908000	0.637133000	3.153190000
Au	-2.296696000	2.729534000	1.472817000
Au	-3.918606000	-2.902337000	-2.989107000
Au	-1.508463000	-1.573912000	4.734554000
Au	-2.639788000	4.769619000	-0.342666000
N	8.018124000	0.132986000	1.951449000
S	6.123915000	-0.085573000	-2.007607000
H	6.093840000	1.237238000	-2.260438000

Sum of electronic and zero-point Energies= -4318.849283
Sum of electronic and thermal Energies= -4318.780935
Sum of electronic and thermal Enthalpies= -4318.779991
Sum of electronic and thermal Free Energies= -4318.982582

[(pc)Ru(NO)(SH)@Au$_{20}$], S$_0$

Ru	7.206371000	-0.002261000	0.657100000
O	8.843124000	0.064003000	3.064255000
C	4.339382000	4.566848000	2.039027000
C	3.143208000	4.789020000	2.715924000
C	2.384596000	3.721679000	3.222703000
C	2.800376000	2.401609000	3.066968000
C	3.997877000	2.177436000	2.391696000
C	4.706635000	0.956435000	2.034994000
N	4.272258000	-0.268593000	2.306423000
C	4.862110000	-1.396723000	1.931221000
C	4.325762000	-2.727505000	2.179762000
C	3.170735000	-3.166028000	2.823634000
C	2.938439000	-4.538445000	2.867542000
C	3.834105000	-5.448347000	2.282943000
C	4.986862000	-5.011990000	1.636678000
C	5.222087000	-3.640617000	1.592186000
C	6.293666000	-2.854959000	0.997171000
N	6.026368000	-1.538368000	1.237637000
N	7.315657000	-3.368497000	0.329254000
C	8.274243000	-2.669541000	-0.261201000
C	9.357138000	-3.252719000	-1.041156000
C	9.687344000	-4.571290000	-1.339242000
C	10.808320000	-4.791065000	-2.134651000
C	11.576293000	-3.722055000	-2.622213000
C	11.245327000	-2.402339000	-2.328609000
C	10.127298000	-2.180514000	-1.530241000
C	9.502309000	-0.958407000	-1.043429000
N	8.425021000	-1.315524000	-0.287242000
N	9.927222000	0.263220000	-1.328730000
C	9.343512000	1.389350000	-0.946176000

C	9.804874000	2.720776000	-1.312011000
C	10.887613000	3.156690000	-2.070361000
C	11.046825000	4.529024000	-2.237467000
C	10.146465000	5.441775000	-1.665305000
C	9.061113000	5.008393000	-0.909729000
C	8.901792000	3.636289000	-0.738302000
C	7.901896000	2.851254000	-0.029023000
N	8.223997000	1.534122000	-0.179995000
N	6.862951000	3.363846000	0.614333000
C	5.922211000	2.665030000	1.231997000
N	5.836787000	1.309457000	1.362669000
C	4.757890000	3.248238000	1.883548000
H	4.927656000	5.389448000	1.645134000
H	2.789131000	5.805611000	2.857857000
H	1.456797000	3.931640000	3.746882000
H	2.209519000	1.574976000	3.450935000
H	2.474294000	-2.460383000	3.267920000
H	2.048185000	-4.914347000	3.363099000
H	3.620410000	-6.511471000	2.337266000
H	5.679484000	-5.712004000	1.180509000
H	9.090042000	-5.395608000	-0.962973000
H	11.095778000	-5.807675000	-2.385236000
H	12.443712000	-3.931463000	-3.240780000
H	11.835416000	-1.573514000	-2.706367000
H	11.580560000	2.448427000	-2.513118000
H	11.881839000	4.903884000	-2.821499000
H	10.301936000	6.505506000	-1.817591000
H	8.362394000	5.711482000	-0.467805000
Au	3.491842000	-0.123050000	-1.111772000
Au	1.065040000	-1.317739000	-1.728473000
Au	1.229948000	1.503957000	-1.210286000
Au	1.610715000	-0.392833000	0.954117000
Au	-0.663368000	1.320422000	0.909685000
Au	-1.261317000	0.318947000	-1.946593000
Au	-0.842096000	-1.694154000	0.365612000
Au	-1.337503000	-2.491354000	-2.314649000
Au	-1.000137000	3.077124000	-1.296914000
Au	-0.258352000	-0.630358000	2.931794000
Au	-3.402954000	0.049136000	0.280563000
Au	-3.416558000	-2.757024000	-0.189628000
Au	-3.822732000	-0.824274000	-2.392655000
Au	-2.902225000	-1.870136000	2.323130000
Au	-3.659250000	1.843557000	-1.906833000
Au	-2.722008000	1.042606000	2.855064000
Au	-3.075813000	2.820437000	0.834599000
Au	-3.867480000	-3.538067000	-2.781597000
Au	-2.279400000	-0.849762000	4.800722000
Au	-3.372406000	4.508180000	-1.310188000
N	8.184529000	0.019501000	2.138140000
S	5.943487000	-0.052923000	-1.455008000
H	6.006391000	1.258466000	-1.751347000

Sum of electronic and zero-point Energies= -4996.750746

Sum of electronic and thermal Energies= -4996.672391

Sum of electronic and thermal Enthalpies= -4996.671447

Sum of electronic and thermal Free Energies= -4996.896512

[(pc)Ru(NO)(SH)@Au_{20}], T_1

Ru	7.075373000	-0.020737000	0.546041000
O	8.394517000	0.976229000	2.908420000
Au	3.453013000	-0.269096000	-1.324196000
Au	0.898398000	-0.842996000	-2.244171000
Au	1.397845000	1.501701000	-0.660269000
Au	1.547966000	-1.144324000	0.530784000
Au	-0.483172000	0.691868000	1.318500000
Au	-1.211658000	1.006592000	-1.683764000
Au	-1.055664000	-1.806201000	-0.391910000
Au	-1.638871000	-1.381323000	-3.137052000
Au	-0.609055000	3.216411000	0.012781000
Au	-0.322234000	-1.953815000	2.351492000
Au	-3.356586000	0.106530000	0.364230000
Au	-3.744621000	-2.238906000	-1.193469000
Au	-3.903201000	0.449337000	-2.403932000
Au	-3.115861000	-2.516132000	1.435229000
Au	-3.395227000	2.650590000	-0.894786000
Au	-2.554281000	-0.115605000	3.084131000
Au	-2.691789000	2.357173000	1.971634000
Au	-4.290124000	-1.844411000	-3.851998000
Au	-2.366103000	-2.670326000	4.074495000
Au	-2.769597000	4.795168000	0.711871000
N	8.039612000	0.120455000	2.195405000
S	5.882241000	-0.121630000	-1.641941000
C	3.750820000	4.045504000	2.370865000
C	2.556131000	4.070485000	3.099599000
C	1.941273000	2.888585000	3.520401000
C	2.499008000	1.637644000	3.229644000
C	3.683203000	1.616454000	2.508938000
C	4.521827000	0.517640000	2.024274000
N	4.245218000	-0.767267000	2.213124000
C	4.975380000	-1.782419000	1.765487000
C	4.607947000	-3.191571000	1.930614000
C	3.515261000	-3.807409000	2.521468000
C	3.468047000	-5.206147000	2.491163000
C	4.482875000	-5.951917000	1.886615000
C	5.582004000	-5.326789000	1.287444000
C	5.626829000	-3.942183000	1.319681000
C	6.594145000	-2.976978000	0.788832000
N	6.153700000	-1.723808000	1.087768000
N	7.690604000	-3.315640000	0.123341000
C	8.560580000	-2.457139000	-0.395516000
C	9.749539000	-2.853934000	-1.154719000
C	10.266279000	-4.094236000	-1.493156000
C	11.443928000	-4.119143000	-2.247985000
C	12.072227000	-2.937007000	-2.646853000
C	11.546366000	-1.686100000	-2.306271000
C	10.381212000	-1.664491000	-1.556680000
C	9.566976000	-0.564272000	-1.033891000
N	8.519755000	-1.098870000	-0.348415000

N	9.839403000	0.720441000	-1.229726000
C	9.100782000	1.733460000	-0.797534000
C	9.413998000	3.142726000	-1.048633000
C	10.457427000	3.758204000	-1.721269000
C	10.453558000	5.156020000	-1.778965000
C	9.434665000	5.902193000	-1.181973000
C	8.381588000	5.277958000	-0.504855000
C	8.389241000	3.893545000	-0.448010000
C	7.467708000	2.929429000	0.159207000
N	7.944829000	1.676859000	-0.077198000
N	6.363383000	3.268674000	0.812252000
C	5.511005000	2.409743000	1.359187000
N	5.582251000	1.051856000	1.358009000
C	4.300340000	2.805603000	2.084102000
H	5.882582000	1.186111000	-1.962657000
H	4.231684000	4.962082000	2.044566000
H	2.100374000	5.024713000	3.345512000
H	1.013230000	2.940660000	4.081924000
H	2.017925000	0.718556000	3.549666000
H	2.722378000	-3.225596000	2.982737000
H	2.627238000	-5.721450000	2.945497000
H	4.416385000	-7.035383000	1.880959000
H	6.369959000	-5.903002000	0.813326000
H	9.774893000	-5.010830000	-1.183198000
H	11.877551000	-5.073636000	-2.529560000
H	12.984608000	-2.990758000	-3.232594000
H	12.031372000	-0.766283000	-2.616713000
H	11.247026000	3.176158000	-2.185398000
H	11.256394000	5.670857000	-2.297426000
H	9.460904000	6.985531000	-1.245363000
H	7.587936000	5.854644000	-0.040949000

Sum of electronic and zero-point Energies= -4996.738554
Sum of electronic and thermal Energies= -4996.659598
Sum of electronic and thermal Enthalpies= -4996.658653
Sum of electronic and thermal Free Energies= -4996.885003

$[(NH_3)_5Ru(NO)]^{3+}$

Ru	-0.081303000	0.001171000	0.005045000
N	1.694705000	-0.001022000	0.012273000
O	2.817612000	0.008106000	-0.003699000
H	-1.143682000	-2.059746000	1.400441000
H	0.342422000	-1.773268000	1.989966000
H	0.164615000	-2.618907000	0.609456000
H	-0.498626000	2.082004000	1.683264000
H	0.678593000	1.159483000	2.316268000
H	-0.873926000	0.718630000	2.494561000
H	0.197938000	1.726459000	-2.060215000
H	0.459091000	2.552421000	-0.690164000
H	-1.063459000	2.239856000	-1.164388000
H	-0.950380000	-1.829744000	-1.772439000
H	0.654275000	-1.672957000	-1.983854000
H	-0.359235000	-0.575042000	-2.626400000
H	-2.640947000	-0.857319000	0.202638000

H	-2.577137000	0.237836000	-0.990043000
H	-2.621410000	0.725347000	0.559913000
N	-0.196833000	-1.142894000	-1.794817000
N	-0.132081000	1.831608000	-1.101131000
N	-0.194136000	-1.814611000	1.123796000
N	-0.213421000	1.114685000	1.825538000
N	-2.208582000	0.028507000	-0.060600000

Sum of electronic and zero-point Energies= -505.590839
Sum of electronic and thermal Energies= -505.578077
Sum of electronic and thermal Enthalpies= -505.577132
Sum of electronic and thermal Free Energies= -505.628004

$[(CN)_5Ru(NO)]^{2-}$

Ru	0.000000000	0.000000000	-0.104154000
C	0.000000000	2.048077000	0.091970000
N	0.000000000	3.208262000	0.224014000
C	2.048077000	0.000000000	0.091970000
N	3.208262000	0.000000000	0.224014000
C	0.000000000	0.000000000	1.951881000
N	0.000000000	0.000000000	3.118268000
C	-2.048077000	0.000000000	0.091970000
N	-3.208262000	0.000000000	0.224014000
C	0.000000000	-2.048077000	0.091970000
N	0.000000000	-3.208262000	0.224014000
N	0.000000000	0.000000000	-1.888701000
O	0.000000000	0.000000000	-3.026892000

Sum of electronic and zero-point Energies= -687.639736
Sum of electronic and thermal Energies= -687.624966
Sum of electronic and thermal Enthalpies= -687.624022
Sum of electronic and thermal Free Energies= -687.681595

Table S2. Major electronic transitions and assignment for the [(L)Ru(NO)(SH)@Au_{20}] complexes calculated at the PBE0/LanL2DZ(Ru)U6-31G(d,p)(E) level in water solvent.

λ (nm)	*f*	**Excitation**	**Assignment**
		**[(salen)Ru(NO)(SH)@Au_{20}]**	
635	0.08	H → L (89%)	MM'CT/MLCT
607	0.04	H → L+1 (88%)	MM'CT/MLCT
491	0.02	H-4 → L+2 (29%), H-3 → L+3 (50%)	MC
		**[(bpb)Ru(NO)(SH)@Au_{20}]**	
612	0.03	H-5 → L+1 (36%), H → L+1 (55%)	LMCT/MC
488	0.03	H-4 → L+3 (42%), H-3 → L+2 (14%), H → L+5 (10%)	MC/MM'CT
		**[(porph)Ru(NO)(SH)@Au_{20}]**	
634	0.05	H-1 → L+1 (30%), HOMO → L+1 (41%)	MM'CT/MLCT
498	0.06	H-1 → L+2 (31%), H-1 → L+4 (17%)	MM'CT/MLCT
484	0.03	HOMO → L+7 (39%)	MC
		**[(pc)Ru(NO)(SH)@Au_{20}]**	
793	0.08	H → L+1 (89%)	MM'CT/MLCT/LL'CT
582	0.55	H-1 → L+3 (80%)	LMCT/IL/LL'CT
548	0.06	H-2 → L+2 (95%)	LMCT/IL/LL'CT

Table S3. %*CT* character of the lowest 30 excited states of [(Salen)Ru(NO)(SH)@Au_{20}]

State	%*CT*[Ru]	%*CT*[Au]	%*CT*[Ru+Au]	Assignment
1	-1.3200	8.5602	7.2402	MLCT
2	-1.4016	31.6335	30.2319	MLCT
3	-1.4485	33.7391	32.2906	MLCT
4	-0.0879	-5.5743	-5.6623	LMCT
5	-0.0217	-5.4717	-5.4935	LMCT
6	-1.2253	17.8589	16.6336	MLCT
7	-1.2252	20.0434	18.8182	MLCT
8	-1.3273	34.0041	32.6769	MLCT
9	-1.1996	27.8924	26.6928	MLCT
10	-1.1702	27.0168	25.8466	MLCT
11	-0.0423	-4.8545	-4.8967	LMCT
12	-0.0973	-0.6013	-0.6986	-
13	-0.9481	24.8501	23.9021	MLCT
14	-0.5727	14.2929	13.7202	MLCT
15	-1.0675	28.3148	27.2473	MLCT
16	-0.7309	18.6089	17.8781	MLCT
17	-0.8247	21.6349	20.8102	MLCT
18	-0.2374	3.0542	2.8169	MLCT
19	-0.2041	3.4715	3.2674	MLCT
20	-0.1883	-0.4124	-0.6007	-
21	-1.0337	26.6189	25.5852	MLCT
22	-0.0484	-1.2424	-1.2909	LMCT
23	-0.1596	2.8024	2.6429	MLCT
24	-0.4681	9.8000	9.3319	MLCT
25	-0.8831	23.5745	22.6914	MLCT
26	-1.3659	38.7122	37.3463	MLCT
27	-0.1274	0.0896	-0.0378	-
28	-0.0144	-4.5944	-4.6088	LMCT
29	-0.1158	-4.4189	-4.5347	LMCT
30	-0.0178	-4.5707	-4.5885	LMCT

Table S4. %*CT* character of the lowest 30 excited states of [(bpb)Ru(NO)(SH)@Au_{20}]

State	%*CT*[Ru]	%*CT*[Au]	%*CT*[Ru+Au]	Assignment
1	-2.2362	2.8375	0.6013	IL/LC
2	-2.4559	30.0548	27.5989	MLCT
3	-1.8524	22.1498	20.2974	MLCT
4	-3.1365	-2.4640	-5.6005	LMCT
5	-1.2505	5.3103	4.0598	MLCT
6	-0.8195	0.1485	-0.6710	-
7	-2.4206	33.5633	31.1426	MLCT
8	-1.0676	4.1087	3.0411	MLCT
9	-2.1022	30.1087	28.0065	MLCT
10	-2.5420	3.0216	0.4796	-
11	-1.5548	0.6250	-0.9298	-
12	-1.8817	0.6544	-1.2273	LMCT
13	-1.0637	-5.6683	-6.7320	LMCT
14	-1.6672	0.1645	-1.5028	LMCT
15	-1.2269	3.3940	2.1671	MLCT
16	-1.9397	25.9486	24.0089	MLCT
17	-1.8110	21.0372	19.2263	MLCT
18	-1.8974	31.6521	29.7547	MLCT
19	-1.8609	14.2434	12.3825	MLCT
20	-1.8714	35.0322	33.1608	MLCT
21	-1.6454	-4.2069	-5.8523	LMCT
22	-0.6653	-7.1295	-7.7948	LMCT
23	-0.4688	13.0789	12.6102	MLCT
24	-1.7652	31.2568	29.4916	MLCT
25	-1.6945	30.2761	28.5816	MLCT
26	0.0010	-2.3914	-2.3905	LMCT
27	-0.0147	-0.8728	-0.8875	LMCT
28	-0.0062	-0.5635	-0.5697	LMCT
29	0.0354	-3.9860	-3.9506	LMCT
30	-0.4676	1.0954	0.6277	MLCT

Table S5. %*CT* character of the lowest 30 excited states of [(porph)Ru(NO)(SH)@Au_{20}]

State	%*CT*[Ru]	%*CT*[Au]	%*CT*[Ru+Au]	Assignment
1	-1.2319	11.0334	9.8015	MLCT
2	-1.2882	12.3277	11.0395	MLCT
3	-1.2701	11.9172	10.6471	MLCT
4	-1.2958	13.8631	12.5673	MLCT
5	-1.1771	6.5937	5.4166	MLCT
6	-1.2235	8.2170	6.9936	MLCT
7	-2.2251	-13.4806	-15.7056	LMCT
8	-1.5858	-10.8055	-12.3912	LMCT
9	-1.6571	-5.6761	-7.3332	LMCT
10	-1.2521	18.7309	17.4788	MLCT
11	-1.1941	1.1128	-0.0813	IL/LC
12	-1.5293	2.6592	1.1299	MLCT
13	-1.3996	20.2622	18.8626	MLCT
14	-0.5799	-18.6754	-19.2553	LMCT
15	-1.1632	16.9154	15.7522	MLCT
16	-1.0838	0.2623	-0.8215	-
17	-0.8536	-10.9488	-11.8024	LMCT
18	-0.9829	6.6716	5.6887	MLCT
19	-1.0613	-0.3407	-1.4019	LMCT
20	-1.3791	16.9864	15.6073	MLCT
21	-1.5760	12.4234	10.8474	MLCT
22	-1.0312	-2.8426	-3.8738	LMCT
23	-1.9779	-3.0944	-5.0723	LMCT
24	-1.2024	-4.2542	-5.4567	LMCT
25	-1.6477	-6.4997	-8.1474	LMCT
26	-0.9400	-7.8427	-8.7827	LMCT
27	-0.5881	-9.3860	-9.9741	LMCT
28	-1.4594	-7.4976	-8.9570	LMCT
29	-1.4408	-11.8282	-13.2690	LMCT
30	-0.5331	-15.0402	-15.5733	LMCT

Table S6. %*CT* character of the lowest 30 excited states of [(pc)Ru(NO)(SH)@Au_{20}]

State	%*CT*[Ru]	%*CT*[Au]	%*CT*[Ru+Au]	Assignment
1	-1.4638	-3.6426	-5.1065	LMCT
2	-1.7319	-2.7153	-4.4472	LMCT
3	-0.8557	25.1503	24.2946	MLCT
4	-1.2995	26.6580	25.3585	MLCT
5	-0.8870	42.1173	41.2303	MLCT
6	-1.4033	43.4939	42.0906	MLCT
7	-0.9246	43.7811	42.8566	MLCT
8	-1.3932	45.0456	43.6525	MLCT
9	-2.2143	23.5765	21.3622	MLCT
10	-0.9376	44.1149	43.1773	MLCT
11	-3.9217	31.5199	27.5983	MLCT
12	-2.5430	38.1651	35.6220	MLCT
13	-1.4101	45.2111	43.8010	MLCT
14	-1.3909	45.2828	43.8919	MLCT
15	-1.9879	-4.7636	-6.7515	LMCT
16	-4.8898	-2.5013	-7.3912	LMCT
17	-0.0330	-14.8694	-14.9024	LMCT
18	0.0536	-16.9281	-16.8745	LMCT
19	-2.1613	39.8906	37.7293	MLCT
20	-0.8750	42.4348	41.5598	MLCT
21	-5.6430	42.9377	37.2947	MLCT
22	-1.3882	43.6887	42.3006	MLCT
23	-2.1109	40.0730	37.9621	MLCT
24	-0.0894	-0.8549	-0.9443	-
25	-1.4692	4.5281	3.0589	MLCT
26	-3.9446	28.6661	24.7215	MLCT
27	-0.1089	-0.6958	-0.8047	-
28	-0.2283	4.5264	4.2982	MLCT
29	-1.6355	31.1478	29.5123	MLCT
30	-1.9844	37.9556	35.9712	MLCT
